# Manipulation of EGFR-Induced Signaling for the Recruitment of Quiescent Neural Stem Cells in the Adult Mouse Forebrain

**DOI:** 10.3389/fnins.2021.621076

**Published:** 2021-03-26

**Authors:** Loïc M. Cochard, Louis-Charles Levros, Sandra E. Joppé, Federico Pratesi, Anne Aumont, Karl J. L. Fernandes

**Affiliations:** ^1^University of Montreal Hospital Research Centre (CRCHUM), Montreal, QC, Canada; ^2^Department of Neurosciences, Faculty of Medicine, Université de Montreal, Montreal, QC, Canada

**Keywords:** epidermal growth factor, neural stem cell, aging, Alzheimer’s disease, neurogenesis, quiescence, electroporation, ventricular-subventricular zone

## Abstract

The ventricular-subventricular zone (V-SVZ) is the principal neurogenic niche in the adult mammalian forebrain. Neural stem/progenitor cell (NSPC) activity within the V-SVZ is controlled by numerous of extrinsic factors, whose downstream effects on NSPC proliferation, survival and differentiation are transduced via a limited number of intracellular signaling pathways. Here, we investigated the relationship between age-related changes in NSPC output and activity of signaling pathways downstream of the epidermal growth factor receptor (EGFR), a major regulator of NSPC activity. Biochemical experiments indicated that age-related decline of NSPC activity *in vivo* is accompanied by selective deficits amongst various EGFR-induced signal pathways within the V-SVZ niche. Pharmacological loss-of-function signaling experiments with cultured NSPCs revealed both overlap and selectivity in the biological functions modulated by the EGFR-induced PI3K/AKT, MEK/ERK and mTOR signaling modules. Specifically, while all three modules promoted EGFR-mediated NSPC proliferation, only mTOR contributed to NSPC survival and only MEK/ERK repressed NSPC differentiation. Using a gain-of-function *in vivo* genetic approach, we electroporated a constitutively active EGFR construct into a subpopulation of quiescent, EGFR-negative neural stem cells (qNSCs); this ectopic activation of EGFR signaling enabled qNSCs to divide in 3-month-old early adult mice, but not in mice at middle-age or carrying familial Alzheimer disease mutations. Thus, (i) individual EGFR-induced signaling pathways have dissociable effects on NSPC proliferation, survival, and differentiation, (ii) activation of EGFR signaling is sufficient to stimulate qNSC cell cycle entry during early adulthood, and (iii) the proliferative effects of EGFR-induced signaling are dominantly overridden by anti-proliferative signals associated with aging and Alzheimer’s disease.

## Introduction

The ventricular-subventricular zone (V-SVZ) of the forebrain lateral ventricles is a highly organized and tightly regulated environment that is permissive for adult neurogenesis (Lim and Alvarez-Buylla, 2016). Resident neural stem cells (NSCs) generate transit amplifying progenitors (TAPs) that undergo several rounds of rapid divisions before in turn giving rise to neuroblasts (Doetsch et al., 1999; Ponti et al., 2013). In the adult rodent brain, thousands of neuroblasts migrate via the rostral migratory stream each day to terminally differentiate into olfactory bulbs neurons (Lois et al., 1996; Doetsch et al., 1999; Alvarez-Buylla and Garcia-Verdugo, 2002; Lim and Alvarez-Buylla, 2016; Obernier and Alvarez-Buylla, 2019). Importantly, neurogenesis in the V-SVZ is responsive to microenvironmental signals (Faigle and Song, 2013; Lim and Alvarez-Buylla, 2016). For example, V-SVZ output can be biased toward oligodendrogenesis under demyelinating conditions (Menn et al., 2006; Xing et al., 2014; Kang et al., 2019) or astrogenesis following traumatic injury (Goings et al., 2004; Benner et al., 2013; Saha et al., 2013), exhibits age-related declines (Enwere et al., 2004; Luo et al., 2006; Ahlenius et al., 2009; Bouab et al., 2011; Paliouras et al., 2012; Shook et al., 2012; Hamilton et al., 2013; Daynac et al., 2016), and can be rejuvenated by circulating systemic factors (Villeda et al., 2011; Katsimpardi et al., 2014).

Integration of the microenvironmental signals that regulate activity of NSCs and TAPs (herein, referred to collectively as neural stem/progenitor cells, NSPCs) is mediated by a limited number of intracellular signaling pathways (Lennington et al., 2003; Faigle and Song, 2013). It is modulation of these intracellular signaling pathways that ultimately controls NSPC survival, proliferation and differentiation (Lim and Alvarez-Buylla, 2016; Cutler and Kokovay, 2020). In particular, signaling pathways modulated via activation of the epidermal growth factor receptor (EGFR) have been particularly implicated in NSPC control (Craig et al., 1996; Kuhn et al., 1997; Doetsch et al., 2002; Faigle and Song, 2013). EGF, the first mitogen identified for NSPCs, enabled NSPCs to be isolated from the rodent brain using neurosphere cultures (Reynolds and Weiss, 1992; Reynolds et al., 1992). RNA sequencing studies revealed that expression of the EGFR is found in rapidly dividing TAPs and in the subpopulation of NSCs that are activated (aNSCs), but not in the upstream quiescent NSCs (qNSCs) (Codega et al., 2014; Llorens-Bobadilla et al., 2015; Dulken et al., 2017). Upon ligand-induced activation, EGFR triggers several signaling cascades that are common to receptor tyrosine kinases (RTKs), such as the PI3K/Akt and Ras/Raf/MEK/ERK MAP kinase pathways, and results in promotion of NSPC survival and proliferation while repressing NSPC differentiation (Reynolds and Weiss, 1992; Annenkov, 2014). Previous studies have shown that mTOR, a downstream target of the PI3K/Akt pathway, is essential during EGFR-mediated NSPC proliferation; mTOR inhibition blocked EGF-induced NSPC proliferation *in vitro* and *in vivo*, and mTOR signaling was necessary for EGF administration to rescue NSPC proliferation in the aging brain (Paliouras et al., 2012; Hartman et al., 2013). However, whether individual downstream branches of EGFR-induced signaling pathways mediate specific aspects of adult NSPC survival, proliferation, and/or differentiation remains unknown.

In the present study, we investigated the contributions and potential of EGFR-triggered signaling pathways for the control of adult NSPC activity in the adult and aging brain. Specifically, we hypothesized that EGFR signaling represents a potential avenue for enhancing NSPC activity in the context of the declining neurogenesis that occurs during normal aging (Eriksson et al., 1998; Curtis et al., 2003; Luo et al., 2006; Shook et al., 2012; Ernst and Frisen, 2015; Daynac et al., 2016) and that is further accelerated in Alzheimer’s disease (AD) (Ziabreva et al., 2006; Hamilton et al., 2010, 2015; Moreno-Jimenez et al., 2019; Scopa et al., 2020). First, we used *in vitro* pharmacological approaches to better understand how the EGFR-triggered AKT, ERK and mTOR signaling cascades coordinate the processes of NSPC survival, proliferation and/or differentiation. We then used an *in vivo* electroporation approach to ectopically express and activate EGFR signaling in a subpopulation of ventricle-contacting qNSCs (Joppe et al., 2020) in models of the early adult, middle-aged, and Alzheimer V-SVZ niche. Altogether, this work provides us with a clearer understanding of the potential for manipulating EGFR-induced signaling pathways for enhancing V-SVZ neurogenesis.

## Results

### Age-Related Modulations of RTK-Induced Signaling Pathways *in vivo*

**FIGURE 1:**
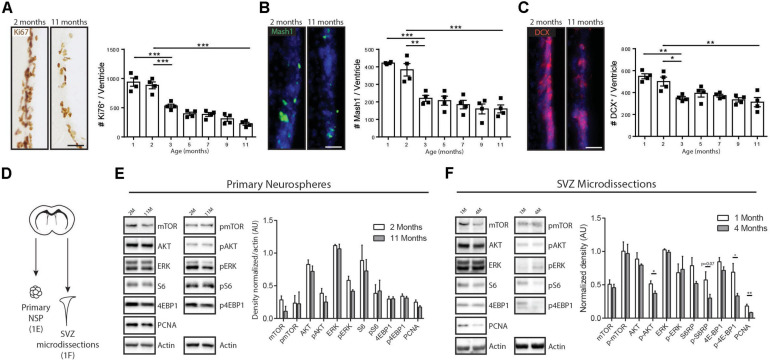
Age-related modulations of neurogenesis and receptor tyrosine kinase-induced signaling pathways. **(A–C)** Kinetics of declining V-SVZ neurogenesis during early- and mid- adulthood. Time course analysis of **(A)** proliferating cells (Ki67 + cells), **(B)** TAPs (Mash1 + cells), and **(C)** Neuroblasts (DCX + cells), with representative pictures and quantifications for each. These cell populations decline sharply between 2 and 3 months of age and then more gradually until 11 months of age (*n* = 4/group). One-way ANOVA with Tukey’s *post hoc* test. **(D)** Paradigm for Western blot analyses. **(E)** Western blots using lysates of primary neurospheres from the V-SVZ of 2- and 11-month-old mice (left) with densitometric quantification (right) (*n* = 3/group). Unpaired *t*-tests. **(F)** Western blots using lysates of microdissected V-SVZ from 1- and 4-month-old mice (left) with densitometric quantification (right) (*n* = 4/group). Unpaired *t*-tests. Scale bar represents 50 μm in **(A–C)**. *p ≤ 0.05; **p ≤ 0.01; ***p ≤ 0.001.

Previous studies have shown that a large component of age-related decreases in V-SVZ neurogenesis actually takes place during the early adulthood to middle-aged period (Bouab et al., 2011; Hamilton et al., 2013; Kalamakis et al., 2019). To more precisely describe the proliferative and neurogenic changes occurring during this period, we used immunohistochemistry to quantify the numbers of Ki67 + proliferating cells, Mammalian achaete-scute homolog-1 (Mash1) + TAPs and Doublecortin (DCX) + neuroblasts in the V-SVZ at multiple ages between juvenile (1-month-old) and middle-aged (11-month-old) timepoints. Overall numbers of Ki67 + proliferating cells declined by 75% over this period (1 month, 938.7 ± 71 Ki67 + cells/SVZ, *n* = 4 versus 11 months, 226 ± 25.2 Ki67 + cells/SVZ, *n* = 4) (Figure 1A), with the sharpest decline occurring between 2 and 3 months of age (40% decline: 2 months, 883.2 ± 59 Ki67 + cells/SVZ, *n* = 4 versus 3 months, 529.5 ± 32.3 Ki67 + cells/SVZ, *n* = 4, *p* < 0.0002, one-way ANOVA). Cell proliferation continued steadily declining at a less pronounced rate between 3 and 11 months (an additional 57.3% decline: 3 months, 529.5 ± 32.3 Ki67 + cells/SVZ, *n* = 4 versus 11 months, 226 ± 25.2 Ki67 + cells/SVZ, *n* = 4, *p* = 0.0015, one-way ANOVA). The changes in cell proliferation were paralleled by similar changes in the Mash1 + progenitor and DCX + neuroblast subpopulations, which likewise showed their sharpest drops between 2 and 3 months of age (Mash1: 42.5% decline between 2 months, 383.1 ± 35.3 cells/SVZ and 3 months, 220.1 ± 17.3 cells/SVZ; DCX: 30% decline between 2 months, 500.5 ± 40.8 cells/SVZ and 3 months, 350.3 ± 14 cells/SVZ; *p* = 0.0018 and *p* = 0.021 respectively, one-way ANOVA, *n* = 4/timepoint) (Figures 1B,C).

Although V-SVZ neurogenesis declined markedly *in vivo* over these timepoints, the intrinsic capacity for NSPCs to activate EGFR-associated signaling pathways remained unchanged. NSPCs cultures were generated from 2- and 11-month-old V-SVZs using EGF-dependent neurosphere cultures (Figure 1D). Analysis of the neurosphere lysates by Western blotting showed equal expression of proliferating cell nuclear antigen (PCNA) and no significant differences in activation levels of multiple downstream components of EGF-induced signaling pathways, including pAKT, pERK, phospho(p)-mTOR, pS6 or p4EBP1 (Figure 1E). Hence, in the presence of exogenous mitogen, NSPCs from the young adult and middle-aged V-SVZ have the same intrinsic capacity to activate EGF-induced AKT, ERK, and mTOR signaling pathways.

We then microdissected the V-SVZ of 1- and 4-month-old animals (C57BL/6, *n* = 4 per age group), bracketing the sharpest period of neurogenesis decline, in order to assess activation of these same signaling molecules *in vivo.* Western blots showed that the V-SVZ of 4-month-old animals had a 55.5% decrease in PCNA expression (1 month, 0.18 ± 0,02 AU versus 4 months, 0.08 ± 0.01 AU, *n* = 4, *p* = 0.007, Unpaired *t*-test) that was accompanied by statistically significant downregulations of pAKT (27.4% decrease between 1 month, 0.51 ± 0.04 AU and 4 months, 0.37 ± 0.03 AU, *n* = 4 per age group, *p* = 0.03, Unpaired *t*-test) and p4EBP1 (51.5% decrease between 1 month, 0.68 ± 0.13 AU and 4 months, 0.33 ± 0.04 AU, *n* = 4 per age group, *p* = 0.03, Unpaired *t*-test) and a strong trend for pS6 (47.4% decrease between 1 month, 0.5731 ± 0.1223 AU and 4 months, 0.30 ± 0.05 AU, *n* = 4 per age group, *p* = 0.07, Unpaired *t*-test) (Figure 1F).

Thus, there is a sharp drop in V-SVZ neurogenesis during the early adulthood period that is accompanied by selective decreases in certain EGF-induced signaling pathways *in vivo*.

### NSPC Activity Is Differentially Regulated by EGF-Induced Signaling Modules

**FIGURE 2:**
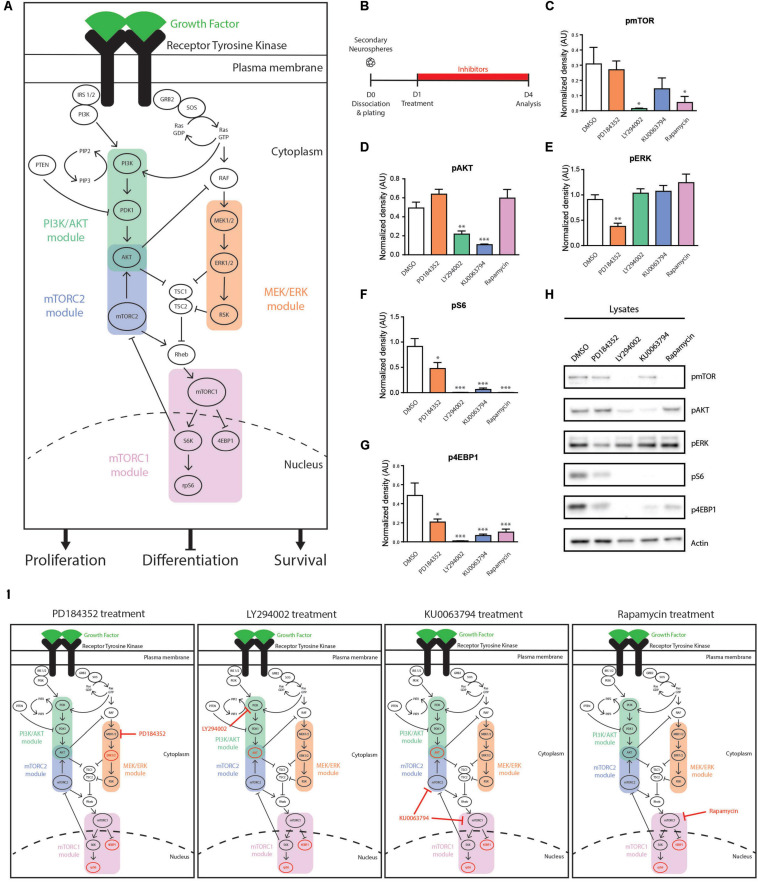
Signaling modules influenced in cultured NSPCs by the pharmacological inhibitors PD184352, LY294002, KU0063794 and Rapamycin. **(A)** Schematic representation of the RTK-induced PI3K/AKT, MEK/ERK, mTORC1, and mTORC2 signaling modules. In NSPCs, activation of these pathways downstream of EGFR result in stimulation of NSPC proliferation and survival and inhibition of NSPC differentiation. **(B–H)** Effect of inhibitors on components of EGF-induced signaling pathways. **(B)** Paradigm. **(C–G)** Densitometric quantifications of the phosphorylated (p) proteins **(C)** pmTOR, **(D)** pAKT, **(E)** pERK, **(F)** pS6, and **(G)** p4EBP1, with **(H)** representative Western blots (*n* = 3/group). One-way ANOVA with Dunnett’s *post hoc* test. **(I)** Summary of signaling modulations induced by each inhibitor. Signaling components with reduced phosphorylation are shown in red. ^∗^*p* ≤ 0.05; ^∗∗^*p* ≤ 0.01; ^∗∗∗^*p* ≤ 0.001.

We performed pharmacological loss-of-function experiments to better understand how modulations of sub-branches of EGFR-induced signaling might impact NSPC proliferation, survival, and differentiation. For these experiments, we focused on two receptor-proximal pathways, the phosphatidylinositol-3-kinase/protein kinase B (PI3K/AKT) and mitogen-activated protein kinase/extracellular-regulated kinase (MAPK/ERK) cascades, and the more downstream mammalian target of rapamycin (mTOR) complexes, mTORC1 and mTORC2 (Figure 2A).

Several widely used pharmacological inhibitors were first assessed for their effects on these individual EGFR signaling modules in NSPCs. Neurospheres were grown the adult V-SVZ and used to generate adherent cultures of adult NSPCs. Based on previous publications and our own pilot studies, NSPC cultures were treated with the PI3K inhibitor LY294002 (25 μM), the MEK1/2 inhibitor PD184352 (10 μM), the mTORC1 inhibitor Rapamycin (40 nM) or the mTORC1/2 inhibitor KU0063794 (10 μM) (Figures 2B,H) (Paliouras et al., 2012; Chen et al., 2015). Western blot analysis showed that (i) LY294002 suppressed the activation of its target PI3K/AKT module (55.88 ± 6.78% decrease of pAKT, DMSO; *n* = 3 per condition) as well as its downstream mTORC1 module (95.49 ± 1.16% decrease of pmTOR, 99 ± 0.15% decrease of pS6 and 97.9 ± 0.47% decrease of p4EBP1; *n* = 3 per condition) (Figures 2C,D,F–H); (ii) PD184352 suppressed activation of its target MEK/ERK module (58.37 ± 6.96% decrease of pERK, DMSO; *n* = 3 per condition) as well as its downstream mTORC1 module (47.83 ± 13.07% decrease of pS6 and 57.16 ± 6.62% decrease of p4EBP1; *n* = 3 per condition) (Figures 2E–H); (iii) Rapamycin suppressed activation of its target mTORC1 module (82.36 ± 13.01% decrease of pmTOR, 99 ± 0.05% decrease of pS6, and 78.96 ± 6.54% decrease of p4EBP1; *n* = 3 per condition) (Figures 2C,F–H); and (iv) KU0063794 suppressed activation of both the mTORC1 and mTORC2 modules (78.46 ± 21.5% decrease of pAKT, 53.16 ± 23.28% decrease of pmTOR, 93.11 ± 3.22% decrease of pS6 and 86.39 ± 3.14% decrease of p4EBP1; *n* = 3 per condition) (Figures 2C,D,F–H). Notably, we observed that phosphorylation of the mTORC1 targets, pS6 and p4EBP1, was more strongly suppressed by the PI3K inhibitor LY294002 than by the MEK inhibitor PD184352 (99 ± 0.15% decrease of pS6 and 97.9 ± 0.47% decrease of p4EBP1 by LY294002 versus 47.83 ± 13.07% decrease of pS6 and 57.16 ± 6.62% decrease of p4EBP1 by PD184352; *n* = 3 per condition), indicating a more important contribution of the PI3K/AKT module than the MEK/ERK module to mTORC1 activity (Figures 2E,F,H). In fact, activation of the mTORC1 module was suppressed at least as much by the PI3K inhibitor LY294002 as by the mTORC1 inhibitor Rapamycin and mTORC1/2 inhibitor KU0063794, highlighting that the PI3K/AKT module is the major driver of EGFR-induced mTOR activity in NSPCs (95.49 ± 1.16%, 82.36 ± 13.01, 53.16 ± 23.28% reductions of pmTOR; 99 ± 0.15%, 99 ± 0.05%, and 93.11 ± 3.22% decreases of pS6; 97.9 ± 0.47%, 78.96 ± 6.54%, and 86.39 ± 3.14% decreases of p4EBP1, induced by LY294002, Rapamycin and KU0063794, respectively; *n* = 3 per condition) (Figures 2C,H). Interestingly, AKT phosphorylation was reduced at least as well by the mTORC1/2 inhibitor KU0063794 as by the PI3K inhibitor LY294002, highlighting the importance of AKT as a target during negative feedback regulation (55.88 ± 6.78% and 78.46 ± 21.5% decreases of pAKT induced by LY294002 and KU0063794, respectively; *n* = 3 per condition) (Figures 2D,H). These findings are summarized in Figure 2I.

**FIGURE 3:**
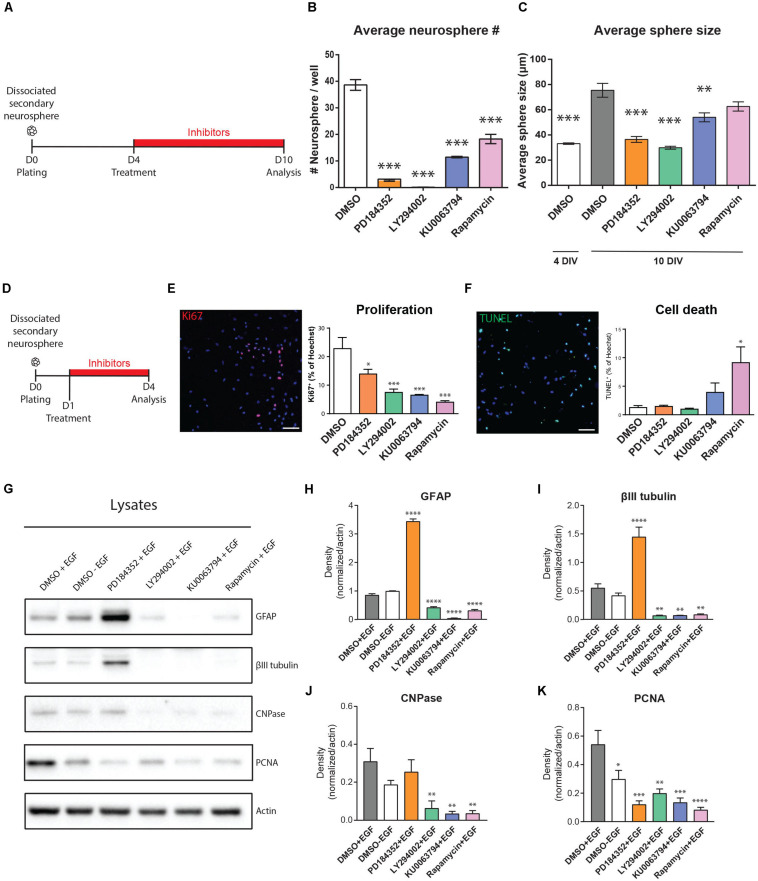
EGF-induced signaling modules show both overlap and selectivity in their regulation of NSPC proliferation, survival, and differentiation. **(A–C)** Effects of inhibitors on neurosphere growth. **(A)** Paradigm for neurosphere formation assay. **(B)** Numbers of neurospheres > 70 microns in size (*n* = 3/group). **(C)** Average size of colonies at the start of treatment (4 DIV) and after 6 days of treatment (10 DIV) (*n* = 3/group). One-way ANOVA with Dunnett’s *post hoc* test for **(B,C)**. **(D–K)** Effects of inhibitors on NSPC proliferation, survival and differentiation. **(D)** Paradigm for adherent NSPC culture assays. **(E)** Representative micrograph of proliferating cells (Ki67, pink) counterstained for total nuclei (Hoechst, blue) with quantification at right (*n* = 3/group). **(F)** Representative micrograph of dying cells (TUNEL, green) counterstained for total nuclei (Hoechst, blue) with quantification at right (*n* = 3/group). One-way ANOVA with Dunnett’s *post hoc* test. **(G)** Representative Western blot of inhibitor-treated NSPCs probed for the astrocytic marker GFAP, neuronal marker βIII-tubulin, oligodendrocytic marker CNPase, proliferation marker PCNA, and loading control Actin, with **(H–K)** their respective densitometric quantifications normalized to Actin (*n* = 4/group). One-way ANOVA with Dunnett’s *post hoc* test. Scale bar represents 80 μm in **(E,F)**. ^∗^*p* ≤ 0.05; ^∗∗^*p* ≤ 0.01; ^∗∗∗^*p* ≤ 0.001; ^∗∗∗^*p* ≤ 0.001; ^****^p ≤ 0.0001.

We first examined the effects of these inhibitors on growth of neurosphere cultures of NSPCs (Figure 3A). When established secondary cultures of neurospheres were dissociated, replated and then treated with the inhibitors from days 4–10 *in vitro* (4DIV–10DIV), formation of tertiary neurospheres was statistically significantly diminished by all inhibitors (Figure 3B). Compared to DMSO treatment (38.63 ± 2 neurosphere/well), the percentage of neurospheres reaching at least 70 microns in diameter was zero with the PI3K inhibitor LY294002, 8.1% with the MEK inhibitor PD184352 (3.16 ± 0.35 neurosphere/well; *n* = 3 per condition), 29.6% with the mTORC1/2 inhibitor KU006394 (11.46 ± 0.27 neurosphere/well; *n* = 3 per condition) and 47.3% with the mTORC1 inhibitor Rapamycin (18.27 ± 1.74 neurosphere/well; *n* = 3 per condition). Although the number of full-size 70 μm neurospheres was reduced by all inhibitors, analysis of the neurosphere size distributions suggested the effects of mTOR inhibition on neurosphere growth involve a different mechanism than the PI3K/AKT and MEK/ERK modules. Treatment of the 4 DIV neurosphere colonies with the PI3K inhibitor LY294002 and MEK inhibitor PD184352 both immediately arrested neurosphere growth at their 4DIV size; in contrast, the average size of neurosphere colonies grown in the presence of the mTOR inhibitors approached the size of the 10DIV DMSO control, with the distribution shifted to a slightly smaller average diameter (DMSO 4D, 32.79 ± 0.61 μm; DMSO 10D, 72.28 ± 2.29 μm; PD184352, 34.7 ± 0.86 μm; LY294002, 29.07 ± 0.64 μm; KU0063794, 56.55 ± 1.50 μm; Rapamycin, 64.15 ± 1.48 μm; *n* = 3 per condition) (Figure 3C).

To explore these observations in greater detail, we used adherent cultures to specifically examine the effects of the inhibitors on NSPC proliferation, survival and differentiation (Figure 3D). Immunocytochemistry for Ki67 expression in inhibitor-treated NSPC cultures showed that MEK inhibition reduced the number of proliferating NSPCs by 39% (from 22.8 ± 3.8% to 13.9 ± 1.6%, *p* = 0.03, one-way ANOVA), while the PI3K, mTORC1, and mTORC1/2 inhibitors reduced NSPC proliferation by 67% (to 7.4 ± 1.1%, *p* = 0.0009, one-way ANOVA), 82% (to 4.04 ± 0.5%, *p* = 0.0002, one-way ANOVA) and 71% (to 6.5 ± 0.3%, *p* = 0.0006, one-way ANOVA), respectively (Figure 3E). The effects of mTORC1 and mTORC1/2 inhibition did not significantly differ. Thus, proliferation of NSPCs is positively regulated by all the PI3K/AKT, MEK/ERK, and mTORC1 modules, with the PI3K/AKT and mTORC1 modules having the stronger contributions. The contribution of all these modules to NSPC proliferation was further supported by Western blotting of culture lysates (Figures 3G,K), which confirms that EGF-induced PCNA expression is significantly reduced in the absence of EGF by 44.93 ± 18.37% (DMSO + EGF, 0.54 ± 0.09 AU versus DMSO-EGF, 0.29 ± 0.06 AU; *n* = 4), and decreases to an even greater extent if EGF-treated NSPCs are with PD184352 (by 77.67 ± 11.57% to 0.12 ± 0.02 AU), LY294002 (by 63.25 ± 4.83% to 0.19 ± 0.03 AU), KU0063794 (by 75.1 ± 5.73% to 0.13 ± 0.03 AU), or Rapamycin (by 84.73 ± 5.88% to 0.08 ± 0.01 AU).

We performed TUNEL assays to determine whether the effects of these inhibitors on NSPC proliferation were secondary to cell death. In contrast to their roles in NSPC proliferation, the PI3K/AKT and MEK/ERK modules did not appear to be key mediators of NSPC survival: 1.2 ± 0.3% of NSPCs were TUNEL + when treated with DMSO control, and this was not statistically affected by treatment with LY294002, PD184352 or KU0063794 (Figure 3F). In contrast, the proportion of TUNEL + cells increased to 9.1 ± 2.7% with the mTORC1 inhibitor Rapamycin (*p* = 0.01, one-way ANOVA), indicating that mTORC1 inhibition, but not PI3K or MEK inhibition, increases sensitivity to cell death.

EGF-treated NSPCs typically fail to exhibit significant differentiation, but it is unclear whether this is due to a direct suppression of differentiation or is simply secondary to the promotion of proliferation. Interestingly, EGF-treated NSPCs treated with the MEK inhibitor PD184352 showed strong increases in both the astrocyte marker GFAP and the neuronal marker βIII-tubulin (Figures 3G–I). This was not observed with LY290042, KU0063794 or Rapamycin, which instead reduced expression of βIII-tubulin, GFAP and the oligodendroglial marker CNPase (Figures 3G–J). We performed immunocytochemistry on NSPCs treated under the same conditions to test whether the changes observed by Western blotting reflected changes in the numbers of cells expressing these markers. Immunocytochemistry confirmed that NSPC cultures yielded tri-lineage differentiation into GFAP + astrocytes, βIII-tubulin + neurons, and CNPase + oligodendrocytes (Supplementary Figure 1A). Upon PD184352 treatment, quantifications revealed a twofold increase of GFAP + cells (DMSO, 35.56 ± 2.70% of Hoechst; PD184352, 73.46 ± 2,87% of Hoechst; *n* = 4 per condition) and fourfold increase of βIII tubulin + cells (DMSO, 14.93 ± 1.62% of Hoechst + cells; PD184352, 62.45 ± 9.59% of Hoechst + cells; *n* = 4 per condition), consistent with the Western blot observations (Supplementary Figures 1B,C). Interestingly, induction of βIII tubulin + cells in the PD184352 condition occurred mainly within the expanded population of GFAP + cells, highlighting transition from a GFAP + precursor at this early 3-day differentiation timepoint. Since NSPC proliferation was reduced by all inhibitors (Figures 3E,K) but differentiation markers were stimulated only by PD184352, only the MEK/ERK module acts as an EGF-induced repressor of NSPC differentiation. Conversely, activity of the PI3K/AKT and mTORC1 modules is necessary for appropriate NSPC differentiation.

These data indicate that the PI3K/AKT, MEK/ERK, and mTOR1/2 modules have both overlap and selectivity in the biological functions of NSPCs that they regulate: all three modules are implicated in NSPC proliferation, the mTORC1 module contributes to NSPC survival and the MEK/ERK module represses NSPC differentiation.

### EGFR-CA Can Promote Neurogenic Activity of qNSCs *in vivo*

Recently, we showed that adult brain electroporation can be used to genetically target a subpopulation of ventricle-contacting, GFAP-expressing cells within the qNSC population (Joppe et al., 2020). In that study, lineage-tracing showed these electroporated precursors produced small numbers of olfactory bulb neurons *in vivo* and did so independently of the neurosphere-forming aNSC lineage. Here, we used this adult electroporation strategy to test whether this highly quiescent qNSC population can be recruited via activation of EGFR-induced signaling.

**FIGURE 4:**
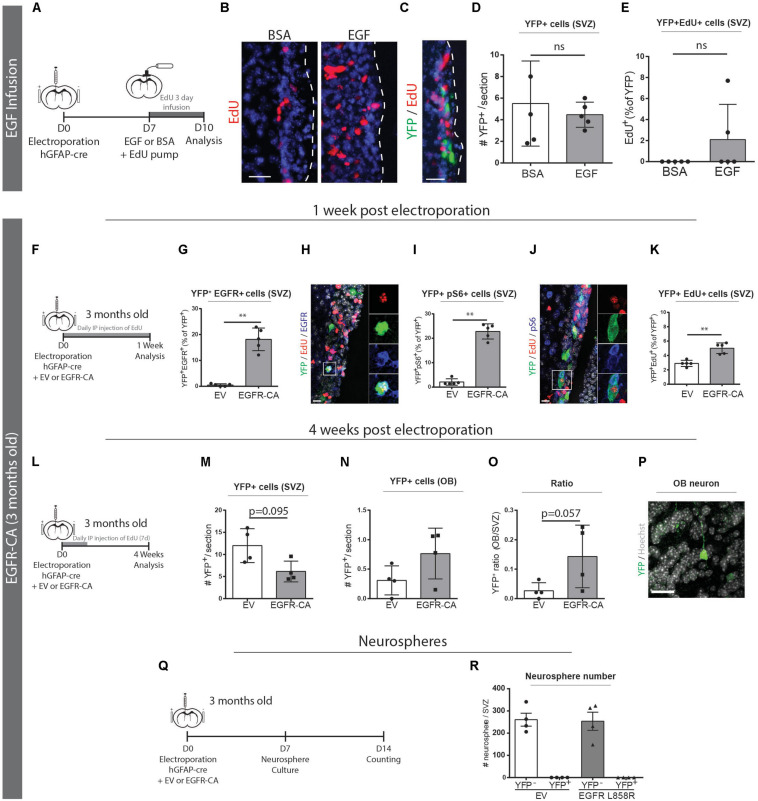
Genetic activation of EGFR signaling can promote qNSC division *in vivo*. **(A–E)** Electroporated GFAP + qNSCs do not divide in response to EGF infusion. **(A)** Paradigm for adult brain electroporation with hGFAP-cre plasmids in Rosa26-stop-EYFP reporter mice followed by 3 days infusion of EdU with either EGF or BSA via intracerebroventricular osmotic pump. **(B)** Representative micrographs of EdU incorporation in the V-SVZ following 3 days infusion of EGF (*n* = 3) or BSA (*n* = 3). **(C)** Representative micrograph and quantifications of **(D)** total YFP + cells and **(E)** YFP + EdU + cells in the V-SVZ. Neither YFP + cells nor YFP + EdU + dividing cells increased in response to EGF infusion. **(F–Q)** EGFR-CA electroporation in 3-month-old mice Rosa26-stop-EYFP mice and tissue analysis after 1-week **(F–K)**, 4-weeks **(L–O)**, or by neurosphere analysis **(P,Q)**. **(F)** 1-week paradigm for adult brain electroporation with hGFAP-cre plasmids, mixed with either EV or EGFR-CA plasmids, in Rosa26-stop-EYFP reporter mice. **(G–K)** Quantifications of the proportions of recombined (YFP+) cells that **(G)** express EGFR protein, **(I)** activate downstream pS6 signaling, and **(K)** incorporate EdU (*n* = 5/group). Shown are representative micrographs of **(H)** YFP/EdU/EGFR and **(J)** YFP/EdU/pS6 immunostainings. **(L)** 4-week paradigm for adult brain electroporation with hGFAP-cre plasmids, mixed with either EV or EGFR-CA plasmids, in Rosa26-stop-EYFP reporter mice. **(M–O)** 4 weeks post-electroporation, quantifications show trends for **(M)** a depletion of YFP + cells in the V-SVZ, **(N)** an increase in YFP + cells in the OB, and **(O)** a consequent increase in the OB/V-SVZ ratio (*n* = 4/group), and **(P)** a representative micrograph of YFP + cell in the OB. **(Q)** Neurosphere formation paradigm for adult brain electroporation with hGFAP-cre plasmids, mixed with either EV or EGFR-CA plasmids, in Rosa26-stop-EYFP reporter mice. **(R)** Quantification of the number of YFP + and YFP- neurospheres produced after 1 month of neurosphere culturing. Note that EGFR-CA electroporation increased qNSC division but did not yield any aNSC-associated recombined neurospheres. Scale bars represent 50 μm in **(B,C)**, and 20 μm in **(H,J)** and 30 μm in **(P)**. Mann–Whitney test, ^∗∗^*p* = 0.0079.

Since qNSCs are reportedly EGFR-negative, we first confirmed that the electroporated subpopulation of qNSCs is not responsive to exogenous EGF. When we implanted 3-day intraventricular osmotic pumps containing EGF, total EdU incorporation in the V-SVZ markedly increased (Figure 4B) but the electroporated cells did not increase in number or show an increase in EdU incorporation (Figures 4C–E).

We then electroporated 3-month-old Rosa26-stop-EYFP reporter mice with hGFAP-cre plasmids that had been mixed with either an empty vector (EV) control or a constitutively active form of EGFR (EGFR-CA) (Figure 4F). To assess the responses of the electroporated qNSCs, we performed fluorescent immunostaining and quantified YFP + cells that were positive for EGFR, pS6 and EdU. At 1 week post-electroporation, the electroporation of EGFR-CA led to a 35-fold increase of electroporated qNSCs expressing the EGFR receptor (EV, 0.5 ± 0.2% of YFP + cells versus EGFR-CA, 18.1 ± 1.9% of YFP^+^ cells; Mann–Whitney, *p* = 0.0079; *n* = 5 per group) (Figures 4G,H). Levels of pS6, which is a downstream readout of EGFR-induced mTOR signaling and a marker of quiescent stem cells that are “primed” for division (Rodgers et al., 2014), was likewise increased (EV, 2.1 ± 0.6% of YFP + cells versus EGFR-CA, 22.8 ± 1.4% of YFP^+^ cells; Mann–Whitney, *p* = 0.0079; *n* = 5 per group) (Figures 4I,J). Notably, EdU incorporation was significantly increased upon EGFR-CA electroporation (EV, 2.9 ± 0.2% of YFP + cells versus EGFR-CA, 5.0 ± 0.3% of YFP + cells; Mann–Whitney, *p* = 0.0079; *n* = 5 per group) (Figure 4K). It is highly unlikely these YFP + EdU + cells incorporated EdU during DNA repair in the absence of division, as control experiments with the DNA repair marker, p-γH2AX, showed that the vast majority of EdU + p-γH2AX + cells in the V-SVZ indeed express the proliferation marker PCNA (98.33 ± 0.65% of EdU + p-γH2AX + cells are PCNA +, *n* = 3) (Supplementary Figure 2). Interestingly, and despite only transient expression of the EGFR-CA plasmid, analysis of a cohort of electroporated mice at 4 weeks post-electroporation revealed trends for EGFR-CA to eventually decrease YFP^+^ cells within the V-SVZ and increase YFP^+^ cells in the OB (Figures 4L–P). In line with this, when we processed electroporated V-SVZs for neurosphere cultures, we found that EGFR-CA-induced activation of qNSCs did not lead to generation of labeled neurospheres (Figures 4Q,R); this is consistent with the electroporated qNSCs being separate from the self-renewing, neurosphere-forming aNSC lineage (Joppe et al., 2020).

These data reveal that genetic activation of EGFR signaling pathways in 3-month-old mice is sufficient to prime qNSCs and shift them into the dividing subpopulation, but these electroporated qNSCs remain distinct from neurosphere-forming aNSCs.

### The Ability of EGFR-CA to Recruit qNSCs Is Overridden by the Contexts of Aging and Alzheimer’s Disease

**FIGURE 5:**
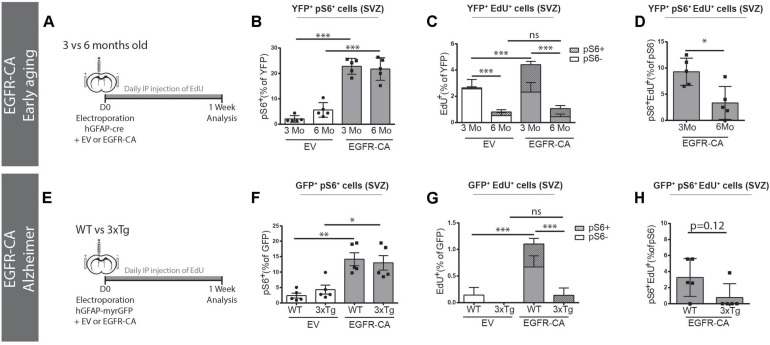
Age and Alzheimer’s disease mutations prevent EGFR-induced qNSCs division. **(A–D)** Baseline and EGFR-CA-induced proliferation of electroporated GFAP + qNSCs cells in 3- versus 6-month-old ROSA26-stop-EYFP mice. **(A)** Paradigm for adult brain electroporation with hGFAP-cre plasmids, mixed with either EV or EGFR-CA plasmids, in Rosa26-stop-EYFP reporter mice. **(B–D)** 1 week post-electroporation, quantification of the percentage of YFP + recombined cells that are **(B)** primed (YFP + pS6+) or **(C)** dividing (YFP + EdU+). Note that YFP + cells in 3- and 6-month-old mice equally upregulated EGFR-induced pS6 but their division significantly increased only in the 3-month-old mice [two way ANOVA: age factor, *F*(1,16) = 141.1, *p* < 0.0001; EGFR-CA factor, *F*(1,16) = 19.62, *p* < 0.0083; interaction, *F*(1,16) = 9.072, *p* = 0.0083]. In **(D)**, the proportion of primed hGFAP + cells that incorporate EdU decreases between 3 and 6 months of age (*p* = 0.0317, Mann–Whitney test). *N* = 5 per condition and age. Three month data is from Figure 4. **(E–H)** Baseline and EGFR-CA-induced proliferation of GFAP + qNSCs in 3xTg-AD mice and their WT control strain. **(E)** Paradigm for adult brain electroporation with hGFAP-GFP plasmids, mixed with either EV or EGFR-CA plasmids, in WT or 3xTg mice. **(F–H)** 1-week post-electroporation, quantification of the percentage of YFP^+^ recombined cells that are **(F)** primed (GFP + pS6+) or **(G)** dividing (GFP + EdU+). Note that GFP + cells in WT and 3xTg mice were equally primed by EGFR-CA but only showed a significant increase in EdU incorporation in the WT mice [two way ANOVA: strain factor, *F*(1,16) = 16.82, *p* = 0.0008; EGFR-CA factor, *F*(1,16) = 16.52, *p* < 0.0009; interaction, *F*(1,16) = 9.245, *p* = 0.0078]. In **(H)**, the proportion of primed GFP + cells that incorporate EdU shows a tendency to decrease in the 3xTg model of Alzheimer’s disease (*p* = 0.12, Mann–Whitney test). *N* = 5 per condition and age. On graphs, white bars represent EV electroporations, gray bars represent EGFR-CA electroporations. In **(C,G)** striped bars represent EdU + pS6+. Two-way ANOVA, ^∗^*p* ≤ 0.05, ^∗∗^*p* ≤ 0.01, ^∗∗∗^*p* ≤ 0.001. Mann–Whitney test, ^∗^*p* = 0.0317.

Lastly, we asked whether EGFR-CA expression in electroporated qNSCs would enable them to overcome the anti-proliferative influences of aging and/or AD on the V-SVZ. When EGFR-CA was overexpressed in 3- versus 6-month-old mice (Figure 5A), electroporated qNSCs showed an equal ability to upregulate EGFR-CA-induced pS6 signaling (3 m EV, 22.8 ± 1.4% versus 6 m EV, 21.7 ± 1.9%, pS6 + YFP + /YFP + cells) (Figure 5B). Interestingly, however, when we examined EdU incorporation, we found that 6-month-old mice had a significantly lower baseline EdU incorporation (3 m EV, 2.9 ± 0.2% versus 6 m EV, 0.4 ± 0.2%, EdU^+^YFP^+^/YFP^+^ cells, *p* < 0.0001, two-way ANOVA) and that, unlike their younger counterparts, the 6-month-old animals failed to show an increase in EdU incorporation in response to EGFR-CA (Figure 5C). Triple co-localization of YFP, EdU, and pS6 confirmed that the increased cell cycle entry observed in 3-month-old mice was restricted to the pS6 + population (Figure 5C) and showed that the propensity of pS6 + cells to incorporate EdU in response to EGFR-CA was decreased at 6 months of age (Figure 5D). Thus, despite retaining the ability to overexpress EGFR and activate downstream signaling, the proportion of hGFAP + qNSCs that enters the cell cycle declines between 3 and 6 months of age and is not rescued by genetic activation of EGFR signaling.

We also analyzed the effects of EGFR-CA overexpression in the 3xTg transgenic mouse model of familial AD, which was developed by Oddo et al. (2003) and carries human familial AD mutations in Presenilin 1, Amyloid Precursor Protein, and Tau. Since 3xTg mice and their WT control strain (B6;129) do not permit Cre-mediated lineage tracing, we used hGFAP-myrGFP reporter plasmids to identify hGFAP + qNSCS cells (Figure 5E). GFP + qNSCs cells of WT and 3xTg mice showed no difference in baseline expression of pS6, and EGFR-CA increased GFP + pS6 + cells equally in both WT and 3xTg mice (Figure 5F). However, an EGFR-CA increase in EdU incorporation was only stimulated in the WT strain (WT EV, 0.1 ± 0.1% versus WT EGFR-CA, 1.1 ± 0.2%, EdU + GFP + /GFP + cells, *p* = 0.0006, two-way ANOVA) (Figure 5G). Consistent with this, the proportion of pS6 + cells that incorporated EdU showed a trend toward a decrease (Figure 5H). Thus, while EGFR-CA can stimulate downstream signaling in qNSCs cells of 3xTg mice, AD-associated signals prevent these cells from undergoing division (Figure 5H).

Together these finding reveal that anti-proliferative signals in the middle-aged and familial AD contexts are dominant over the ability of EGFR-CA to promote qNSC division.

## Discussion

**FIGURE 6:**
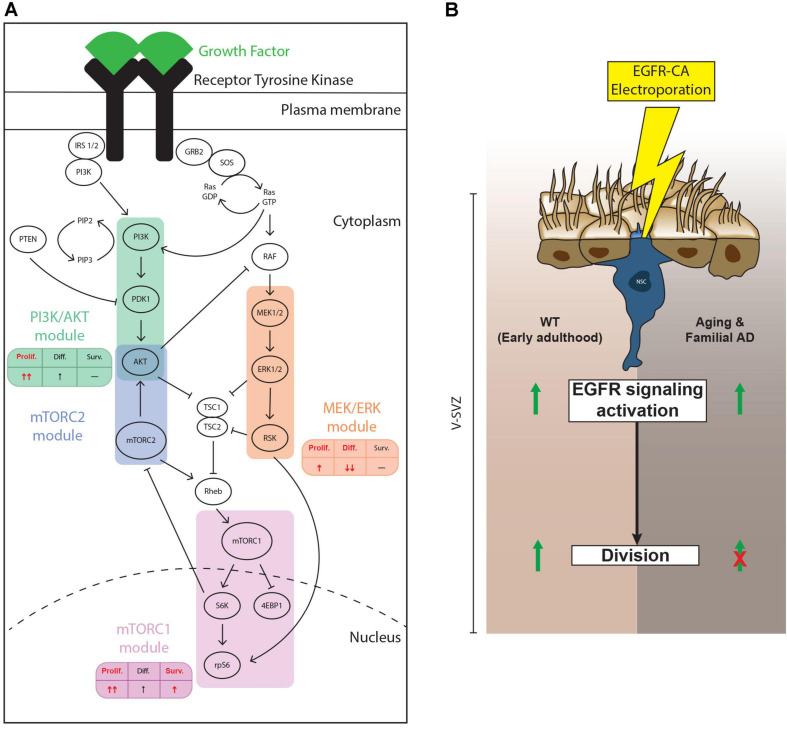
Summary of findings. **(A)** Schematic showing the findings from our pharmacological loss-of-function experiments. *In vitro*, the PI3K/AKT, mTORC1/2 and, to a lesser extent, MEK/ERK, modules all contribute to EGF-induced NSPC proliferation. Only the mTORC1/2 modules showed evidence of contributing to NSPC survival, while only the MEK/ERK module mediated EGF-induced repression of NSPC differentiation. The effect of mTORC1 and mTORC1/2 modules did not significantly differ for these parameters, so we present only mTORC1. In each table, the main effect of each module is represented in red. **(B)** Effect of ectopic activation of EGFR-induced signaling in electroporated qNSCs. In young adult mice (left side of diagram), electroporation with EGFR-CA upregulates levels of pS6, a downstream marker of EGFR signaling and of “primed” qNSCs (Rodgers et al., 2014) and promotes their cell cycle entry. EGFR-CA electroporation in qNSCs of mice at middle age or in mice carrying familial Alzheimer’s disease (AD) mutations (right side of diagram) remains sufficient to activate EGFR-induced pS6, but these qNSCs do not undergo cell division. Thus, the antiproliferative signals associated with aging and AD are dominant to the pro-proliferative effects of EGFR-induced signaling pathways.

Neural stem/progenitor cells in the adult V-SVZ are continuously exposed to a variety of niche signals, which modulate neurogenic output by regulating proliferation, differentiation, and survival of these precursor cells (Lim and Alvarez-Buylla, 2016). Transduction of niche signals occurs via multiple families of receptors, such as RTKs, Eph receptors, interleukin receptors and G-protein coupled receptors for example, which ultimately converge onto a relatively limited number of intracellular signaling pathways (Obernier and Alvarez-Buylla, 2019). Here, we focused on EGFR and three of its downstream signaling pathways that are shared with other RTKs: the PI3K/AKT, MEK/ERK and mTOR cascades (Oda et al., 2005; Lemmon and Schlessinger, 2010; Annenkov, 2014). Our findings support 3 main conclusions. First, NSPCs from the young adult and middle-aged V-SVZ are equally able to activate these pathways in culture but show differences *in vivo*. Second, the PI3K/AKT, MEK/ERK and mTOR signaling cascades exhibit differential impacts on NSPC proliferation, survival and differentiation. Third, ectopic expression of an activated EGFR receptor in qNSCs stimulates their activation and division in the young adult brain, but aging and AD mutations dominantly suppress EGFR-triggered qNSC division (Figures 6A,B).

### Activation of RTK-Induced Signaling Pathways in NSPCs During Early- and Mid-Adulthood

Age-related declines in adult neurogenesis occur in virtually all mammals examined, including humans (Maslov et al., 2004; Luo et al., 2006; Bouab et al., 2011; Ernst et al., 2014; Boldrini et al., 2018). Our time-course analysis revealed a biphasic decline of neurogenesis within the V-SVZ of C57Bl6 mice: a steep slope of decline in proliferation, TAPs and neuroblasts until approximately 3 months of age followed by a more gradual rate of decline until the latest 11-month timepoint. Declining neurogenesis is likely to result from changes to both the intrinsic properties of aging NSPCs and extrinsic properties of the aging niche (Hamilton et al., 2013; Capilla-Gonzalez et al., 2015; Lupo et al., 2019). Our comparison of the response of NSPCs from 2- and 11- month-old mice to EGF supports the idea that the intrinsic proliferative ability of NSPCs is maintained over this period (Ahlenius et al., 2009; Bouab et al., 2011; Shook et al., 2012; Apostolopoulou et al., 2017). Notably, however, multiple downstream components of the mTORC1 and mTORC2 pathways were selectively decreased in the V-SVZ niche between 1 and 4 months of age (pAKT and p4EBP1, with a strong trend toward decrease of pS6). Interestingly, recent studies have implicated mTOR in the regulation of lysosome function in NSPCs, with lysosome-mediated elimination of protein aggregates being necessary and sufficient for NSCs to transition from the quiescent to activated state (Leeman et al., 2018; Morrow et al., 2020). It remains to be demonstrated whether the observed impairments in mTOR signaling are causally related to age-related reductions in proteostasis (Leeman et al., 2018).

### PI3K/AKT, MEK/ERK, and mTOR Signaling Cascades Differentially Regulate NSPC Proliferation, Survival, and Differentiation

We used a pharmacological loss-of-function approach to study the individual contributions of the PI3K/AKT, MEK/ERK, and mTOR pathways to proliferation, survival, and differentiation in NSPC cultures. In control experiments, we verified that there was no cross inhibition between the PI3K inhibitor, LY294002, and the MEK inhibitor, PD184352. Besides their own signaling modules, these inhibitors also both inhibited the downstream mTOR pathway, but to different extents: MEK inhibited partially and PI3K inhibited completely the phosphorylation of mTORC1 targets. NSPC proliferation in response to EGF, as measured by Ki67 staining, was compromised by all four of the inhibitors, indicating that the PI3K/AKT, MEK/ERK, and mTORC1 signaling modules are all important in this process. Rapamycin and KU0063794 had equivalent effects, suggesting that mTORC2 signaling is not involved. Previous studies have also found that NSPC proliferation was inhibited by Rapamycin (Paliouras et al., 2012; Wang et al., 2016), LY294002, or U0126, another inhibitor of MEK/ERK signaling (Yuan et al., 2015).

Interestingly, when the inhibitors were used in neurosphere cultures, neurosphere growth was completely arrested by the PI3K inhibitor, LY294002, and the MEK inhibitor, PD184352, and only partially affected by the mTOR inhibitors, suggesting different modes of action. This may be related to our finding that only the mTOR inhibitors promoted cell death. This was surprisingly, given that mTOR signaling was also repressed upon PI3K inhibition, and may be due to the fact that the PI3K/AKT module has other targets besides mTOR. MEK/ERK inhibition did not increase cell death and, thus, is dispensable for NSPC survival. Our data indicate that only the mTOR pathway is involved in the process of EGF-mediated NSPC survival.

Differentiation in NSPC cultures is normally blocked in the presence of EGF. However, treatment with PD184352 significantly increased the expression of the differentiation markers GFAP and βIII tubulin. This effect was not observed by inhibiting either the PI3K/AKT or mTOR modules, suggesting that EGF-induced repression of NSPC differentiation is mediated specifically by the MEK/ERK signaling module. A previous study reported a similar observation with embryonic NSPCs cultured from the E15 telencephalon, where NSPCs treated with U126 showed reduced proliferation and increased neuronal differentiation (Wang et al., 2009); however, in that study, MEK/ERK inhibition reduced astrocytic differentiation, which was strongly increased here.

We conclude that the PI3K/AKT, MEK/ERK and mTOR1/2 modules play overlapping but dissociable roles in the biological effects of EGF on NSPCs: all 3 modules are implicated in NSPC proliferation (with the PI3K/AKT and mTOR modules being prominent), only the mTORC1 module contributes to NSPC survival and only the MEK/ERK module represses NSPC differentiation.

### Activation of EGFR Signaling Pathways to Promote qNSC Activation

Since NSPC proliferation required activity of all three branches of EGFR signaling that we examined, we tested whether upregulation of EGFR signaling would be sufficient to trigger proliferation of qNSCs *in vivo*. We recently found that a population of quiescent, ventricle-contacting neural precursors that produces small numbers of olfactory bulb neurons can be genetically targeted by adult brain electroporation (Joppe et al., 2020). We therefore used this approach to transfect these quiescent precursors in 3-month-old adult male mice with a constitutively active EGFR construct (EGFR-CA). This led to detectable EGFR protein expression, and downstream mTOR expression in 20–25% of the electroporated qNSCs. After 1 week, their proliferation was increased, and after 1 month, they showed a trend for an increase in labeled cells within the OB. Thus, EGFR-CA is sufficient to recruit these quiescent precursors and potentially increase their neurogenic output in the young adult V-SVZ.

V-SVZ neurogenesis declines with age and this is further reduced in models of AD, including 3xTg mice (Hamilton et al., 2010, 2015). We therefore tested whether forced intrinsic expression of EGFR-CA would be sufficient to enable NSPCs to overcome the inhibitory influence of age and/or AD. Since neurogenesis in the V-SVZ is markedly decreased between 3 and 6 months of age, we first electroporated 3- versus 6-month-old wildtype mice with EGFR-CA. While electroporated mice of both ages increased expression of the mTORC1 readout, pS6, only electroporated cells in the 3-month-old mice increased their proliferation. Similarly, when we electroporated 3xTg mice and their strain controls, both increased pS6 expression but only the strain controls increased proliferation. Thus, activation of EGFR-induced signaling pathways promotes the activation and proliferation of qNSCs, but this is insufficient to allow qNSCs to overcome the inhibitory effects of age or AD on proliferation.

Previous studies have reported that aging in the V-SVZ is associated with a reduced expression of pro-mitotic factors such as FGF-2 and VEGF (Shetty et al., 2005; Bernal and Peterson, 2011). Conversely, there is also an increase of anti-proliferative factors from microglia (Solano Fonseca et al., 2016), astrocytes (Clarke et al., 2018), and the blood (Villeda et al., 2011; Katsimpardi et al., 2014). Our findings suggest declining neurogenesis during normal or pathological aging is primarily due to the presence of anti-proliferative factors present in the microenvironment rather than a loss of mitogens and indicate that such anti-proliferative factors are dominant over the effects of EGFR-induced signaling pathways.

### Conclusion

In order to utilize the neurogenic capacity of adult NSPCs to prevent and/or reverse the age-related defects in the brain, it is essential to better understand the relationship between intrinsic and environmental mechanisms that control their activity. In this work, we show that EGFR-mediated activation of the PI3K/AKT, MEK/ERK, and mTOR cascades play dissociable roles in the control of NSPC proliferation, differentiation and survival *in vitro*. Forced activation of EGFR signaling can recruit quiescent NSPCs into division *in vivo* but is not by itself able to overcome dominant antiproliferative signals associated with normal or Alzheimer-type aging. This suggests that optimal NSPC recruitment during aging is likely to be achieved only through combined NSPC stimulation and neutralization of antiproliferative extrinsic factors.

## Materials and Methods

### Contact for Reagent and Resource Sharing

Further information and requests for resources and reagents should be directed to and will be fulfilled by the Lead Contact, KF (karl.fernandes@usherbrooke.ca).

### Experimental Model and Subject Details

Animal work was conducted in accordance with the guidelines of the Canadian Council of Animal Care and were approved by the animal care committees of the University of Montreal and the Research Center of the University of Montreal Hospital (CRCHUM) (protocol N16002KFs, approval from January 2016). We used 42 male C57BL/6 (C57BL/6NTac, obtained from Taconic) for the time course experiment cultures and microdissections, 45 male Rosa26-stop-EYFP [B6.19 × 1-Gt(ROSA)26Sor^*tm1*(EYFP)Cos^/J, stock number: 006148, obtained from the Jackson Laboratory] for the infusion, electroporation and culture experiments, 10 female 3xTg-AD [B6;129-Psen1^*tm1Mpm*^ Tg(APPSwe,tauP301L)1Lfa/Mmjax; stock number: 034830-JAX, obtained from the Jackson Laboratory] and 10 female of their wildtype control strain (B6129SF2/J; stock number: 101045, obtained from the Jackson Laboratory) for electroporation experiments. Mice were socially housed (up to 5 mice/cage) on a 12 h light-dark cycle with free access to water and food.

### Method Details

#### Surgical Procedures

Mice were provided with water supplemented with acetaminophen as an analgesic (1.34 mg/ml, Tylenol) for 4 days, starting 1 day prior to surgery (Foley et al., 2019), in accordance with the animal committee requirements (protocol N16002KFs, approval from January 2016). Surgeries were performed under isoflurane general anesthesia (Baxter) and Bupivacaine local anesthesia (1 mg/kg, Hospira).

##### Electroporation

For the electroporations in young adults, we used 3-month old male Rosa26-stop-EYFP mice (*n* = 5 per condition). For the electroporations followed by neurosphere cultures experiment, we used 3-month old male Rosa26-stop-EYFP mice (*n* = 4 per group). For the electroporation in early middle-aged animals, we used 6-month old male Rosa26-stop-EYFP mice (*n* = 5 per condition). For the electroporation in an Alzheimer model, we used 6-month old female 3xTg mice and their WT control strain (*n* = 5 per group). Adult brain electroporation was conducted as described previously (Barnabe-Heider et al., 2008; Joppe et al., 2015; Joppe et al., 2020). Plasmids (Supplementary Table 1) were amplified by using an endotoxin-free 40 min Fast Plasmid Maxiprep Kit (Biotool), then purified and concentrated by ethanol precipitation. Intracerebroventricular plasmid injections were performed using a 10 μl Hamilton syringe into the left ventricle at coordinates: 0 mm anteroposterior (AP), +0.9 mm mediolateral (ML), –1.5 mm dorsoventral (DV) to Bregma, using a stereotaxic apparatus (Stoelting Co.), based on The Mouse Brain in Stereotaxic Coordinates, Compact 3rd Edition (Academic Press). Animals received an ICV injection of 10 μg of total DNA in 2 μl, delivered over 2 min, following by five pulses at 50 ms intervals at 200 V applied with 7 mm platinum Tweezertrodes (Havard Apparatus) and an electroporator (ECM 830, Havard Apparatus). Plasmids are listed in Supplementary Table 1.

##### Osmotic pump infusions

For the electroporation followed by osmotic pump infusion experiment, we used 3-month old male Rosa26-stop-EYFP (*n* = 5 per condition). Mice were under isoflurane general anesthesia (1% oxygen, 2% isoflurane) during the whole procedure. For electroporations, intracerebroventricular plasmid injections were performed using a 10 μl Hamilton syringe into the left ventricle at coordinates: 0 mm anteroposterior (AP), +0.9 mm mediolateral (ML), –1.5 mm dorsoventral (DV) to Bregma using a stereotaxic apparatus (Stoelting Co.), based on The Mouse Brain in Stereotaxic Coordinates, Compact 3rd Edition (Academic Press). Immediately after the electroporation, we proceeded with the ICV infusions. The ICV infusions were performed using 3-day osmotic pumps (Alzet, model 1003D, Durect) attached to brain infusion cannulae (Alzet, Brain infusion kit 3, Durect). Pump cannulae were implanted contralateral to the electroporation (right ventricle) procedure using a stereotaxic apparatus (Stoelting Co.) at coordinates: 0 mm anteroposterior (AP), –0.9 mm mediolateral (ML), –1.5 mm dorsoventral (DV) to Bregma, based on The Mouse Brain in Stereotaxic Coordinates, Compact 3rd Edition (Academic Press). Cannulae were fixed to the skull using dental cement (Co-oral-ite Dental Mfg). Osmotic pumps were filled with EGF diluted in a vehicle solution (0.1% BSA in PBS) and infused at 400 ng/day for 3 days, then animals were euthanized by intraperitoneal injection of ketamine/xylazine (347/44 mg/kg, Bimeda-MTC/Boehringer Ingelheim Canada Ltd) for immediate analysis.

#### Tissue Analysis

Mice were euthanized by intraperitoneal injection of ketamine/xylazine (347/44 mg/kg, Bimeda-MTC/Boehringer Ingelheim Canada Ltd). For immunostaining in the time-course (Figure 1), we used C57BL/6 mice (*n* = 28) and for electroporation experiments (Figures 4, 5) we used Rosa26-stop-EYFP (*n* = 36), WT (*n* = 10), and 3xTg (*n* = 10) mice. For these experiments, mice were intracardially perfused with PBS (Wisent) followed by freshly prepared 4% paraformaldehyde (Acros). Brains were removed, post-fixed overnight, and then cut into 40 μm sections using a Leica VT1000S vibrating microtome. Tissue sections were stored in antifreeze at –20°C (Bouab et al., 2011). For neurosphere assays (Rosa26-stop-EYFP, *n* = 3/condition) and V-SVZ microdissections (C57BL/6, *n* = 4/age group), brains were instead dissected from freshly euthanized mice.

For the biochemical analysis of 1- vs. 4- month old V-SVZ, we used microdissections from C57BL/6 (*n* = 4/age group) (Figure 1), microdissected V-SVZ and striatal samples were obtained from freshly dissected mouse brains as follows. Brains were placed in a brain mold and straight-edge razor blades were used to cut a 2-mm-thick coronal section through the forebrain (corresponding to the region between 3- and 5-mm posterior to the anterior edge of the olfactory bulbs). Using a dissecting microscope, fine-tipped tungsten needles were then used to dissect out a block of V-SVZ tissue, by first making lateral cuts through the corpus callosum (dorsally) and above the anterior commissures (ventrally), and then tracing the gray-white boundaries at the V-SVZ/striatum and V-SVZ/septum borders. The V-SVZ region was defined by the cell-dense tissue directly located around the ventricle. The Striatum region was defined by its location adjacent to the ventricle and its fibrous aspect. A block of striatal tissue of similar size was cut from the center of the adjacent striatum for biochemical comparison and to provide a control for SVZ microdissection purity. A schematic of the microdissection procedure is presented in Supplementary Figure 3.

##### Immunostaining

Antibodies are listed in Supplementary Table 2. Immunostaining was performed as described previously (Bouab et al., 2011; Gregoire et al., 2014) on 40 μm-thick sections. 5–6 sections taken every 240 μm, along the AP axis of the brains were used for the stainings. For immunohistochemical labeling, free-floating sections were washed in PBS, and blocked for 2 h with 0.1% Triton X-100 (Fisher Scientific) in PBS supplemented with 4% bovine serine albumin (BSA, Jackson ImmunoResearch, West Grove, PA, United States). Primary antibodies were applied at room temperature overnight in 0.1% Triton X-100 in PBS containing 2% BSA. For fluorescence detection, we used secondary antibodies conjugated to either CY3 (1:1000, Jackson ImmunoResearch) or Alexa 488, 555, or 647 (1:1000, Invitrogen, Burlington, ON, Canada) diluted in PBS for 45 min at room temperature, and nuclei were counterstained with Hoechst 33342 (0.2 μM, Sigma) for 2 min. Citrate-EDTA antigen retrieval was used for immunostaining with DCX and Ki67 antibodies. This method was used for sections from time-course and electroporation experiments, using C57BL/6 (*n* = 28) or Rosa26-stop-EYFP (*n* = 36), WT (*n* = 10), and 3xTg (*n* = 10) mice, respectively.

EdU (5-ethynyl-2′-deoxyuridine) staining was performed as described by Salic and Mitchison (2008). Briefly, sections were incubated in EdU reaction solution (100 mM Tris-buffered saline, 2 mM CuSO_4_, 4 μM Sulfo-Cyanine 3 Azide, and 100 mM Sodium Ascorbate) for 5 min, then washed with two quick wash before to be incubated 5 min in copper blocking reaction (10 mM THPTA in PBS). When EdU staining was coupled with another antibody staining, EdU was performed first. This method was used for the sections from the electroporation experiments using Rosa26-stop-EYFP (*n* = 36), WT (*n* = 10), and 3xTg mice (*n* = 10).

##### Western blotting

Cultures analyzed by Western blotting were lysed in Ripa Buffer as previously described (Bouab et al., 2011). Protein samples for Western blotting were prepared as described previously (Bouab et al., 2011; Paliouras et al., 2012), and primary antibody information is provided in Supplementary Table 2. Ten micrograms of protein from each sample were run per lane. Membranes were blocked for 2 h with 5% milk in TBST. Primary antibodies diluted in TBST were applied at 4°C overnight. Secondary antibodies were applied at room temperature for 45 min and washed in TBST. Signals were revealed using the Clarity kit (Bio-Rad), detected using ChemiDoc (Bio-Rad). Membranes were subsequently stripped with Re-Blot Plus Mild (Millipore), reblocked, and reprobed appropriately. Quantifications were performed using Image Lab 4.1 software (Bio-Rad). Normalized densities were obtained by taking the ratio of the optical density of the protein of interest and dividing by the optical density of Actin. This method was used for the 1 vs. 4 months experiments using C57BL/6 (*n* = 4/age group), 2 vs. 11 months experiments using Rosa26-stop-EYFP (*n* = 3/group) and cell culture experiments using Rosa26-stop- EYFP (*n* = 3–4/group).

##### Cell culture experiments

For the neurosphere treated with inhibitors, we used 3-month old male Rosa26-stop-EYFP mice, *n* = 3/4 per condition. Neurosphere cultures were generated from adult mouse striatum using 20 ng/ml EGF (Sigma) and a protocol based on Reynolds and Weiss (Reynolds and Weiss, 1992) as detailed previously (Gregoire et al., 2014; Hamilton et al., 2015; Joppe et al., 2015). Briefly, both striata, including their associated SVZs, were dissected into ice cold HBSS (Wisent), dissociated to single cells using papain (Worthington, Lakewood, NJ, United States), diluted to 30 ml in a neural stem cell proliferation medium, and then seeded into 75 cm^2^ flasks (BD Bioscience, Mississauga, ON, Canada). Neural stem cell proliferation medium consisted of Dulbecco’s Modified Eagle’s Medium (DMEM)/F12 (3:1) (both from Invitrogen) supplemented with 1% Penicillin/Streptomycin (Wisent), 1 μg/ml Fungizone (Invitrogen), 2% B27 (Invitrogen), 20 ng/ml epidermal growth factor (EGF) (Sigma). For the signaling experiments, we used additional inhibitors that included PD184352 (Sigma), LY294002 (Tocris Bioscience), Rapamycin (Invitrogen), KU0063794 (Tocris Bioscience), as indicated. Cultures of primary neurospheres were passaged by mechanical dissociation and were expanded at a clonal plating density of 2000 cells/cm2. Secondary/tertiary neurospheres were used when it was necessary to amplify neural precursor numbers for high-density adherent cultures for biochemical analysis.

For Ki67/TUNEL and GFAP/(II-tubulin/CNPase immunostainings, secondary neurospheres were dissociated and plated in 8-well chamber slides, at 25,000 cells/cm^2^ in medium containing 20 ng/ml EGF. After 24 h (1 DIV), cells were washed twice with DMEM/F12 and then placed in medium containing 20 ng/ml EGF supplemented with either LY294002 (25 μM), PD184352 (10 μM), KU0063794 (10 μM) or Rapamycin (40 nM) for an additional 3 days. Cells were processed for immunolabeling after 3 days of treatment (4DIV). For this method, we used Rosa26-stop-EYFP mice (*n* = 3–4/group).

For differentiation experiments, secondary neurospheres were dissociated and plated at 25,000 cells/cm^2^ in medium containing 20 ng/ml EGF. After 24 h (1 DIV), cells were washed twice with DMEM/F12 and then placed in medium containing 20 ng/ml EGF supplemented with either LY294002 (25 μM), PD184352 (10 μM), KU0063794 (10 μM) or Rapamycin (40 nM) for an additional 3 days. Cells were lysed after 3 days of treatment (4DIV) for Western blotting. For this method, we used Rosa26-stop-EYFP mice (*n* = 3/group).

For the experiments comparing the effects of treatment with PD184352, LY294002, KU0063497 and Rapamycin on neurosphere number and size, secondary neurospheres were dissociated and plated at 1,500 cells/ml in medium containing 20 ng/ml EGF. The inhibitors LY294002 (25 μM), PD184352 (10 μM), KU0063794 (10 μM), or Rapamycin (40 nM) were added on the day of plating (0 DIV) or 4 days after plating (4 DIV). Neurospheres were counted and measured after 10 DIV. For this method, we used Rosa26-stop-EYFP mice (*n* = 3/group).

Neurosphere numbers were quantified by plating cells in 24-well plates at the clonal densities indicated above (minimum of 8 wells/treatment/N). Neurosphere sizes were quantified by measuring the diameter of at least 100 neurospheres/condition using Fiji software (version 1.52p v, NIH, United States) and graphed using GraphPad Prism, Version 8.4 (GraphPad Software, Inc).

### Quantification and Statistical Analyses

Immunostained tissue sections were examined using a motorized Olympus IX81 fluorescence microscope, an Olympus BX43F light microscope, or a TCS-SP5 inverted microscope (Leica Microsystems, Exton, United States). All quantifications were performed by a blinded observer using coded slides and 40X or 60X objectives. For quantification of total Ki67, Mash1, DCX, EGFR, or p-S6 cells, 3–6 V-SVZ sections/animal were analyzed. For quantification of electroporated cells and their progeny, 6–12 sections/animal were used for the V-SVZ and 5–15 sections/animal for the OB. Counts in the V-SVZ were limited to the DAPI-defined subventricular zone. Counts in the OB (Figures 4N,O) were performed by scanning the OB sections for positive cells at 32x objective magnification. All YFP positive cells in the V-SVZ or OB were confirmed for the presence of a DAPI-stained nucleus. No electroporated animals were excluded from this study.

All statistical analyses were achieved using GraphPad Prism, Version 8.4.3 (GraphPad Software, Inc). The statistical analyses that were performed are indicated in the respective figure legends and included either parametric tests (two-tailed unpaired Student’s *t*-test, one-way or two-way ANOVA with Tukey’s or Dunnett’s post-test) or the non-parametric Mann–Whitney test (the latter in the case of numbers of electroporated cells, which might follow a non-normal distribution). Error bars represent mean ± standard error of the mean (SEM). Significance level was set at *p* ≤ 0.05.

## Data Availability Statement

The original contributions presented in the study are included in the article/Supplementary Material, further inquiries can be directed to the corresponding author/s.

## Ethics Statement

The animal study was reviewed and approved by Animal Care Committees of the University of Montreal and the Research Center of the University of Montreal Hospital (CRCHUM).

## Author Contributions

LC and KF: conceptualization. LC, SJ, and L-CL: methodology. LC, SJ, L-CL, FP, and AA: investigation. LC and KF: manuscript writing and visualization. KF: supervision and funding acquisition. All authors contributed to the article and approved the submitted version.

## Conflict of Interest

The authors declare that the research was conducted in the absence of any commercial or financial relationships that could be construed as a potential conflict of interest.

## Supplementary Material

Supplementary Figure 1 **(A)** Representative micrograph of a triple-labeled NSPC culture co-stained for GFAP (first micrograph), βIII tubulin (second micrograph), CNPase + (third micrograph) and merged (fourth micrograph). **(B)** Representative micrographs of NSPC cultures stained with GFAP (astrocytes), βIII tubulin (neurons) and CNPase (oligodendrocytes) upon different treatments. **(C–E)** Quantification of the proportion of Hoechst-labeled cells that are positive for **(C)** GFAP, **(D)** βIII tubulin, and **(E)** CNPase. Scale bars represent 50 μm in **(A,B)**.

Supplementary Figure 2 **(A)** Representative micrograph of EdU/p-γH2AX/PCNA staining in the V-SVZ. **(B)** Quantifications of the p-γH2AX cells labelled with EdU. **(C)** Quantification of the p-γH2AX + EdU + cells that express PCNA + (actively dividing). Note that virtually all EdU + p-γH2AX + cells are PCNA+. Scale bar represents 50 μm in **(A)**.

Supplementary Figure 3 **(A)** Schematic view of the brain slice used for SVZ and striatum microdissections. The outer rectangle represents the brain mold and the lines with arrows represent the site of sectioning with razor blades. **(B)** Representative micrographs of the slice obtained as seen from the rostral side (left micrograph) and caudal side (right micrograph). The blue dotted square (a) represents region collected for the striatum and the green dotted shape (b) represents the region collected for the V-SVZ.

Supplementary Table 1 Plasmid list.

Supplementary Table 2 Antibody list.

## Abbreviations

AD: Alzheimer’s disease
AKT: protein kinase B
DCX: doublecortin
DMSO: dimethyl sulfoxide
EdU: 5-Ethynyl-2′-deoxyuridine
EGF: epidermal growth factor
EGFR: epidermal growth factor receptor
ERK: extracellular signal-regulated kinase
4EBP1: eukaryotic translation initiation factor 4E-binding protein 1
FGF: fibroblast growth factor
FGFR: fibroblast growth factor receptor
GFAP: glial fibrillary acidic protein
MAPK (MEK): mitogen-activated protein kinase
Mash1: Mammalian achaete-scute homolog-1
mTOR: Mammalian target of rapamycin
NSC: neural stem cell
aNSC: activated neural stem cell
qNSC: quiescent neural stem cell
NSPC: neural stem/progenitor cell
PCNA: proliferating cell nuclear antigen
PI3K: phosphoinositide 3-kinase
RMS: rostral migratory stream
RTK: receptor tyrosine kinase
Sox2: sex determining region Y-box 2
S6: rpS6 for ribosomal protein S6
TAP: transit amplifying progenitor
TUNEL: terminal deoxynucleotidyl transferase dUTP nick end labeling
V-SVZ: ventricular-subventricular zone
VEGF: vascular endothelial growth factor
YFP: yellow fluorescent protein.

## References

[B1] Ahlenius H. Visan V. Kokaia M. Lindvall O. Kokaia Z. (2009). Neural stem and progenitor cells retain their potential for proliferation and differentiation into functional neurons despite lower number in aged brain. *J. Neurosci.* 29 4408–4419. 10.1523/JNEUROSCI.6003-08.2009 19357268 PMC6665731

[B2] Alvarez-Buylla A. Garcia-Verdugo J. M. (2002). Neurogenesis in adult subventricular zone. *J. Neurosci.* 22 629–634. 10.1523/JNEUROSCI.22-03-00629.2002 11826091 PMC6758521

[B3] Annenkov A. (2014). Receptor tyrosine kinase (RTK) signalling in the control of neural stem and progenitor cell (NSPC) development. *Mol. Neurobiol.* 49 440–471. 10.1007/s12035-013-8532-5 23982746

[B4] Apostolopoulou M. Kiehl T. R. Winter M. Cardenas, De La Hoz E. Boles N. C. (2017). Non-monotonic changes in progenitor cell behavior and gene expression during aging of the adult V-SVZ neural stem cell niche. *Stem Cell Rep.* 9 1931–1947. 10.1016/j.stemcr.2017.10.005 29129683 PMC5785674

[B5] Barnabe-Heider F. Meletis K. Eriksson M. Bergmann O. Sabelstrom H. Harvey M. A. (2008). Genetic manipulation of adult mouse neurogenic niches by in vivo electroporation. *Nat. Methods* 5 189–196. 10.1038/nmeth.1174 18204459

[B6] Benner E. J. Luciano D. Jo R. Abdi K. Paez-Gonzalez P. Sheng H. (2013). Protective astrogenesis from the SVZ niche after injury is controlled by Notch modulator Thbs4. *Nature* 497 369–373. 10.1038/nature12069 23615612 PMC3667629

[B7] Bernal G. M. Peterson D. A. (2011). Phenotypic and gene expression modification with normal brain aging in GFAP-positive astrocytes and neural stem cells. *Aging Cell* 10 466–482. 10.1111/j.1474-9726.2011.00694.x 21385309 PMC3094510

[B8] Boldrini M. Fulmore C. A. Tartt A. N. Simeon L. R. Pavlova I. Poposka V. (2018). Human hippocampal neurogenesis persists throughout aging. *Cell Stem Cell* 22:e585. 10.1016/j.stem.2018.03.015 29625071 PMC5957089

[B9] Bouab M. Paliouras G. N. Aumont A. Forest-Berard K. Fernandes K. J. (2011). Aging of the subventricular zone neural stem cell niche: evidence for quiescence-associated changes between early and mid-adulthood. *Neuroscience* 173 135–149. 10.1016/j.neuroscience.2010.11.032 21094223

[B10] Capilla-Gonzalez V. Lavell E. Quinones-Hinojosa A. Guerrero-Cazares H. (2015). Regulation of subventricular zone-derived cells migration in the adult brain. *Adv. Exp. Med. Biol.* 853 1–21. 10.1007/978-3-319-16537-0_1 25895704

[B11] Chen H. Guo R. Zhang Q. Guo H. Yang M. Wu Z. (2015). Erk signaling is indispensable for genomic stability and self-renewal of mouse embryonic stem cells. *Proc. Natl. Acad. Sci. U S A.* 112 E5936–E5943. 10.1073/pnas.1516319112 26483458 PMC4640739

[B12] Clarke L. E. Liddelow S. A. Chakraborty C. Munch A. E. Heiman M. Barres B. A. (2018). Normal aging induces A1-like astrocyte reactivity. *Proc. Natl. Acad. Sci. U S A.* 115 E1896–E1905. 10.1073/pnas.1800165115 29437957 PMC5828643

[B13] Codega P. Silva-Vargas V. Paul A. Maldonado-Soto A. R. Deleo A. M. Pastrana E. (2014). Prospective identification and purification of quiescent adult neural stem cells from their in vivo niche. *Neuron* 82 545–559. 10.1016/j.neuron.2014.02.039 24811379 PMC4360885

[B14] Craig C. G. Tropepe V. Morshead C. M. Reynolds B. A. Weiss S. van der Kooy D. (1996). In vivo growth factor expansion of endogenous subependymal neural precursor cell populations in the adult mouse brain. *J. Neurosci.* 16 2649–2658. 10.1523/JNEUROSCI.16-08-02649.1996 8786441 PMC6578757

[B15] Curtis M. A. Penney E. B. Pearson A. G. van Roon-Mom W. M. Butterworth N. J. Dragunow M. (2003). Increased cell proliferation and neurogenesis in the adult human Huntington’s disease brain. *Proc. Natl. Acad. Sci. U S A.* 100 9023–9027. 10.1073/pnas.1532244100 12853570 PMC166431

[B16] Cutler R. R. Kokovay E. (2020). Rejuvenating subventricular zone neurogenesis in the aging brain. *Curr. Opin. Pharmacol.* 50 1–8. 10.1016/j.coph.2019.10.005 31756641 PMC7234918

[B17] Daynac M. Morizur L. Chicheportiche A. Mouthon M. A. Boussin F. D. (2016). Age-related neurogenesis decline in the subventricular zone is associated with specific cell cycle regulation changes in activated neural stem cells. *Sci. Rep.* 6:21505. 10.1038/srep21505 26893147 PMC4759590

[B18] Doetsch F. Caille I. Lim D. A. Garcia-Verdugo J. M. Alvarez-Buylla A. (1999). Subventricular zone astrocytes are neural stem cells in the adult mammalian brain. *Cell* 97 703–716. 10.1016/S0092-8674(00)80783-7 10380923

[B19] Doetsch F. Petreanu L. Caille I. Garcia-Verdugo J. M. Alvarez-Buylla A. (2002). EGF converts transit-amplifying neurogenic precursors in the adult brain into multipotent stem cells. *Neuron* 36 1021–1034. 10.1016/S0896-6273(02)01133-9 12495619

[B20] Dulken B. W. Leeman D. S. Boutet S. C. Hebestreit K. Brunet A. (2017). Single-Cell transcriptomic analysis defines heterogeneity and transcriptional dynamics in the adult neural stem cell lineage. *Cell Rep.* 18 777–790. 10.1016/j.celrep.2016.12.060 28099854 PMC5269583

[B21] Enwere E. Shingo T. Gregg C. Fujikawa H. Ohta S. Weiss S. (2004). Aging results in reduced epidermal growth factor receptor signaling, diminished olfactory neurogenesis, and deficits in fine olfactory discrimination. *J. Neurosci.* 24 8354–8365. 10.1523/JNEUROSCI.2751-04.2004 15385618 PMC6729689

[B22] Eriksson P. S. Perfilieva E. Bjork-Eriksson T. Alborn A. M. Nordborg C. Peterson D. A. (1998). Neurogenesis in the adult human hippocampus. *Nat. Med.* 4 1313–1317. 10.1038/3305 9809557

[B23] Ernst A. Alkass K. Bernard S. Salehpour M. Perl S. Tisdale J. (2014). Neurogenesis in the striatum of the adult human brain. *Cell* 156 1072–1083. 10.1016/j.cell.2014.01.044 24561062

[B24] Ernst A. Frisen J. (2015). Adult neurogenesis in humans- common and unique traits in mammals. *PLoS Biol.* 13:e1002045. 10.1371/journal.pbio.1002045 25621867 PMC4306487

[B25] Faigle R. Song H. (2013). Signaling mechanisms regulating adult neural stem cells and neurogenesis. *Biochim. Biophys. Acta* 1830 2435–2448. 10.1016/j.bbagen.2012.09.002 22982587 PMC3541438

[B26] Foley P. L. Kendall L. V. Turner P. V. (2019). Clinical management of pain in rodents. *Comp. Med.* 69 468–489. 10.30802/AALAS-CM-19-000048 31822323 PMC6935704

[B27] Goings G. E. Sahni V. Szele F. G. (2004). Migration patterns of subventricular zone cells in adult mice change after cerebral cortex injury. *Brain Res.* 996 213–226. 10.1016/j.brainres.2003.10.034 14697499

[B28] Gregoire C. A. Bonenfant D. Le Nguyen A. Aumont A. Fernandes K. J. (2014). Untangling the influences of voluntary running, environmental complexity, social housing and stress on adult hippocampal neurogenesis. *PLoS One* 9:e86237. 10.1371/journal.pone.0086237 24465980 PMC3900491

[B29] Hamilton L. K. Aumont A. Julien C. Vadnais A. Calon F. Fernandes K. J. (2010). Widespread deficits in adult neurogenesis precede plaque and tangle formation in the 3xTg mouse model of Alzheimer’s disease. *Eur. J. Neurosci.* 32 905–920. 10.1111/j.1460-9568.2010.07379.x 20726889

[B30] Hamilton L. K. Dufresne M. Joppe S. E. Petryszyn S. Aumont A. Calon F. (2015). Aberrant lipid metabolism in the forebrain niche suppresses adult neural stem cell proliferation in an animal model of Alzheimer’s Disease. *Cell Stem Cell* 17 397–411. 10.1016/j.stem.2015.08.001 26321199

[B31] Hamilton L. K. Joppe S. E. Cochard L. M. Fernandes K. J. (2013). Aging and neurogenesis in the adult forebrain: what we have learned and where we should go from here. *Eur. J. Neurosci.* 37 1978–1986. 10.1111/ejn.12207 23773067

[B32] Hartman N. W. Lin T. V. Zhang L. Paquelet G. E. Feliciano D. M. Bordey A. (2013). mTORC1 targets the translational repressor 4E-BP2, but not S6 kinase 1/2, to regulate neural stem cell self-renewal in vivo. *Cell Rep.* 5 433–444. 10.1016/j.celrep.2013.09.017 24139800

[B33] Joppe S. E. Cochard L. M. Levros L. C. Jr. Hamilton L. K. Ameslon P. (2020). Genetic targeting of neurogenic precursors in the adult forebrain ventricular epithelium. *Life Sci. All.* 3:e202000743. 10.26508/lsa.202000743 32482782 PMC7266992

[B34] Joppe S. E. Hamilton L. K. Cochard L. M. Levros L. C. Aumont A. Barnabe-Heider F. (2015). Bone morphogenetic protein dominantly suppresses epidermal growth factor-induced proliferative expansion of adult forebrain neural precursors. *Front. Neurosci.* 9:407. 10.3389/fnins.2015.00407 26576147 PMC4625077

[B35] Kalamakis G. Brune D. Ravichandran S. Bolz J. Fan W. Ziebell F. (2019). Quiescence modulates stem cell maintenance and regenerative capacity in the aging brain. *Cell* 176:e1414. 10.1016/j.cell.2019.01.040 30827680

[B36] Kang W. Nguyen K. C. Q. Hebert J. M. (2019). Transient redirection of SVZ stem cells to oligodendrogenesis by FGFR3 activation promotes remyelination. *Stem Cell Reports* 12 1223–1231. 10.1016/j.stemcr.2019.05.006 31189094 PMC6565886

[B37] Katsimpardi L. Litterman N. K. Schein P. A. Miller C. M. Loffredo F. S. Wojtkiewicz G. R. (2014). Vascular and neurogenic rejuvenation of the aging mouse brain by young systemic factors. *Science* 344 630–634. 10.1126/science.1251141 24797482 PMC4123747

[B38] Kuhn H. G. Winkler J. Kempermann G. Thal L. J. Gage F. H. (1997). Epidermal growth factor and fibroblast growth factor-2 have different effects on neural progenitors in the adult rat brain. *J. Neurosci.* 17 5820–5829. 10.1523/JNEUROSCI.17-15-05820.1997 9221780 PMC6573198

[B39] Leeman D. S. Hebestreit K. Ruetz T. Webb A. E. McKay A. Pollina E. A. (2018). Lysosome activation clears aggregates and enhances quiescent neural stem cell activation during aging. *Science* 359 1277–1283. 10.1126/science.aag3048 29590078 PMC5915358

[B40] Lemmon M. A. Schlessinger J. (2010). Cell signaling by receptor tyrosine kinases. *Cell* 141 1117–1134. 10.1016/j.cell.2010.06.011 20602996 PMC2914105

[B41] Lennington J. B. Yang Z. Conover J. C. (2003). Neural stem cells and the regulation of adult neurogenesis. *Reprod. Biol. Endocrinol.* 1:99. 10.1186/1477-7827-1-99 14614786 PMC293430

[B42] Lim D. A. Alvarez-Buylla A. (2016). The adult ventricular-subventricular zone (V-SVZ) and Olfactory Bulb (OB) neurogenesis. *Cold Spring Harb. Perspect. Biol.* 8:a018820. 10.1101/cshperspect.a018820 27048191 PMC4852803

[B43] Llorens-Bobadilla E. Zhao S. Baser A. Saiz-Castro G. Zwadlo K. Martin-Villalba A. (2015). Single-Cell transcriptomics reveals a population of dormant neural stem cells that become activated upon brain injury. *Cell Stem Cell* 17 329–340. 10.1016/j.stem.2015.07.002 26235341

[B44] Lois C. Garcia-Verdugo J. M. Alvarez-Buylla A. (1996). Chain migration of neuronal precursors. *Science* 271 978–981. 10.1126/science.271.5251.978 8584933

[B45] Luo J. Daniels S. B. Lennington J. B. Notti R. Q. Conover J. C. (2006). The aging neurogenic subventricular zone. *Aging Cell* 5 139–152. 10.1111/j.1474-9726.2006.00197.x 16626393

[B46] Lupo G. Gioia R. Nisi P. S. Biagioni S. Cacci E. (2019). Molecular mechanisms of neurogenic aging in the adult mouse subventricular zone. *J. Exp. Neurosci.* 13:1179069519829040. 10.1177/1179069519829040 30814846 PMC6381424

[B47] Maslov A. Y. Barone T. A. Plunkett R. J. Pruitt S. C. (2004). Neural stem cell detection, characterization, and age-related changes in the subventricular zone of mice. *J. Neurosci.* 24 1726–1733. 10.1523/JNEUROSCI.4608-03.2004 14973255 PMC6730468

[B48] Menn B. Garcia-Verdugo J. M. Yaschine C. Gonzalez-Perez O. Rowitch D. Alvarez-Buylla A. (2006). Origin of oligodendrocytes in the subventricular zone of the adult brain. *J. Neurosci.* 26 7907–7918. 10.1523/JNEUROSCI.1299-06.2006 16870736 PMC6674207

[B49] Moreno-Jimenez E. P. Flor-Garcia M. Terreros-Roncal J. Rabano A. Cafini F. Pallas-Bazarra N. (2019). Adult hippocampal neurogenesis is abundant in neurologically healthy subjects and drops sharply in patients with Alzheimer’s disease. *Nat. Med.* 25 554–560. 10.1038/s41591-019-0375-9 30911133

[B50] Morrow C. S. Porter T. J. Xu N. Arndt Z. P. Ako-Asare K. Heo H. J. (2020). Vimentin coordinates protein turnover at the aggresome during neural stem cell quiescence exit. *Cell Stem Cell* 26 558–568e559. 10.1016/j.stem.2020.01.018 32109376 PMC7127969

[B51] Obernier K. Alvarez-Buylla A. (2019). Neural stem cells: origin, heterogeneity and regulation in the adult mammalian brain. *Development* 146:dev156059. 10.1242/dev.156059 30777863 PMC6398449

[B52] Oda K. Matsuoka Y. Funahashi A. Kitano H. (2005). A comprehensive pathway map of epidermal growth factor receptor signaling. *Mol. Syst. Biol.* 1:2005.0010. 10.1038/msb4100014 16729045 PMC1681468

[B53] Oddo S. Caccamo A. Shepherd J. D. Murphy M. P. Golde T. E. Kayed R. (2003). Triple-transgenic model of Alzheimer’s disease with plaques and tangles: intracellular abeta and synaptic dysfunction. *Neuron* 39 409–421. 10.1016/S0896-6273(03)00434-3 12895417

[B54] Paliouras G. N. Hamilton L. K. Aumont A. Joppe S. E. Barnabe-Heider F. Fernandes K. J. (2012). Mammalian target of rapamycin signaling is a key regulator of the transit-amplifying progenitor pool in the adult and aging forebrain. *J. Neurosci.* 32 15012–15026. 10.1523/JNEUROSCI.2248-12.2012 23100423 PMC6704835

[B55] Ponti G. Obernier K. Guinto C. Jose L. Bonfanti L. Alvarez-Buylla A. (2013). Cell cycle and lineage progression of neural progenitors in the ventricular-subventricular zones of adult mice. *Proc. Natl. Acad. Sci. U S A.* 110 E1045–E1054. 10.1073/pnas.1219563110 23431204 PMC3600494

[B56] Reynolds B. A. Tetzlaff W. Weiss S. (1992). A multipotent EGF-responsive striatal embryonic progenitor cell produces neurons and astrocytes. *J. Neurosci.* 12 4565–4574. 10.1523/JNEUROSCI.12-11-04565.1992 1432110 PMC6575989

[B57] Reynolds B. A. Weiss S. (1992). Generation of neurons and astrocytes from isolated cells of the adult mammalian central nervous system. *Science* 255 1707–1710. 10.1126/science.1553558 1553558

[B58] Rodgers J. T. King K. Y. Brett J. O. Cromie M. J. Charville G. W. Maguire K. K. (2014). mTORC1 controls the adaptive transition of quiescent stem cells from G0 to G(Alert). *Nature* 510 393–396. 10.1038/nature13255 24870234 PMC4065227

[B59] Saha B. Peron S. Murray K. Jaber M. Gaillard A. (2013). Cortical lesion stimulates adult subventricular zone neural progenitor cell proliferation and migration to the site of injury. *Stem Cell Res.* 11 965–977. 10.1016/j.scr.2013.06.006 23900166

[B60] Salic A. Mitchison T. J. (2008). A chemical method for fast and sensitive detection of DNA synthesis in vivo. *Proc. Natl. Acad. Sci. U S A.* 105 2415–2420. 10.1073/pnas.0712168105 18272492 PMC2268151

[B61] Scopa C. Marrocco F. Latina V. Ruggeri F. Corvaglia V. La Regina F. (2020). Impaired adult neurogenesis is an early event in Alzheimer’s disease neurodegeneration, mediated by intracellular Abeta oligomers. *Cell Death Differ.* 27 934–948. 10.1038/s41418-019-0409-3 31591472 PMC7206128

[B62] Shetty A. K. Hattiangady B. Shetty G. A. (2005). Stem/progenitor cell proliferation factors FGF-2, IGF-1, and VEGF exhibit early decline during the course of aging in the hippocampus: role of astrocytes. *Glia* 51 173–186. 10.1002/glia.20187 15800930

[B63] Shook B. A. Manz D. H. Peters J. J. Kang S. Conover J. C. (2012). Spatiotemporal changes to the subventricular zone stem cell pool through aging. *J. Neurosci.* 32 6947–6956. 10.1523/JNEUROSCI.5987-11.2012 22593063 PMC3359841

[B64] Solano Fonseca R. Mahesula S. Apple D. M. Raghunathan R. Dugan A. Cardona A. (2016). Neurogenic niche microglia undergo positional remodeling and progressive activation contributing to age-associated reductions in neurogenesis. *Stem Cells Dev.* 25 542–555. 10.1089/scd.2015.0319 26857912 PMC4817564

[B65] Villeda S. A. Luo J. Mosher K. I. Zou B. Britschgi M. Bieri G. (2011). The ageing systemic milieu negatively regulates neurogenesis and cognitive function. *Nature* 477 90–94. 10.1038/nature10357 21886162 PMC3170097

[B66] Wang B. Gao Y. Xiao Z. Chen B. Han J. Zhang J. (2009). Erk1/2 promotes proliferation and inhibits neuronal differentiation of neural stem cells. *Neurosci. Lett.* 461 252–257. 10.1016/j.neulet.2009.06.020 19539699

[B67] Wang X. Seekaew P. Gao X. Chen J. (2016). Traumatic brain injury stimulates neural stem cell proliferation via mammalian target of rapamycin signaling pathway activation. *eNeuro* 3:ENEURO.0162-16.2016. 10.1523/JNEUROSCI.3491-13.2014 27822507 PMC5089538

[B68] Xing Y. L. Roth P. T. Stratton J. A. Chuang B. H. Danne J. Ellis S. L. (2014). Adult neural precursor cells from the subventricular zone contribute significantly to oligodendrocyte regeneration and remyelination. *J. Neurosci.* 34 14128–14146. 10.1523/JNEUROSCI.3491-13.2014 25319708 PMC6705285

[B69] Yuan H. Chen R. Wu L. Chen Q. Hu A. Zhang T. (2015). The regulatory mechanism of neurogenesis by IGF-1 in adult mice. *Mol. Neurobiol.* 51 512–522. 10.1007/s12035-014-8717-6 24777577

[B70] Ziabreva I. Perry E. Perry R. Minger S. L. Ekonomou A. Przyborski S. (2006). Altered neurogenesis in Alzheimer’s disease. *J. Psychosom. Res.* 61 311–316. 16938507 10.1016/j.jpsychores.2006.07.017

