# Supplementary figures and images for: Manipulation of EGFR-Induced Signaling for the Recruitment of Quiescent Neural Stem Cells in the Adult Mouse Forebrain
Source: Front Neurosci. 2021 Mar 26;15:621076. doi: 10.3389/fnins.2021.621076 (PMC8032885; doi:10.3389/fnins.2021.621076)

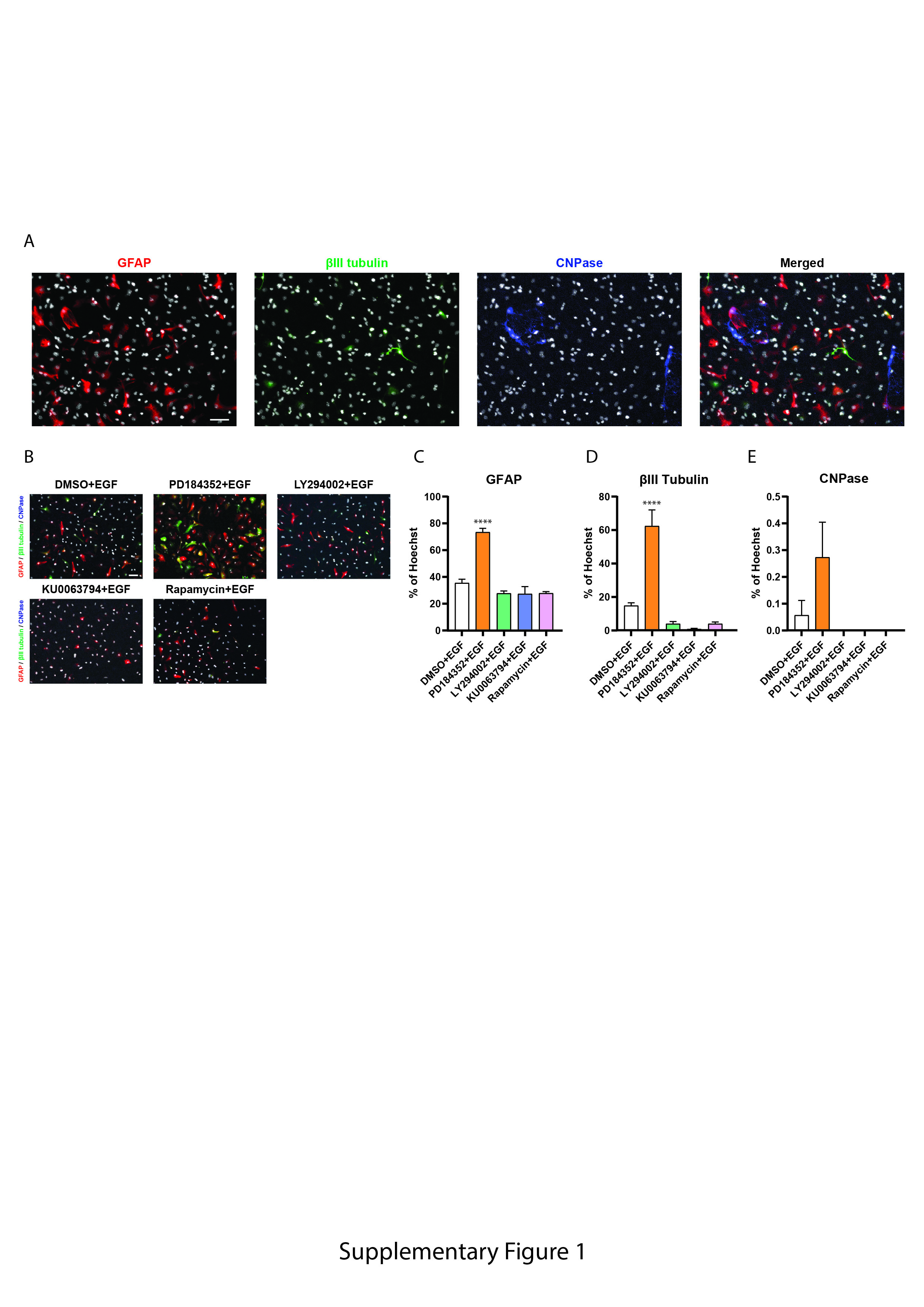

Supplement: Supplementary Figure 1 — (A) Representative micrograph of a triple-labeled NSPC culture co-stained for GFAP (first micrograph), βIII tubulin (second micrograph), CNPase + (third micrograph) and merged (fourth micrograph). (B) Representative micrographs of NSPC cultures stained with GFAP (astrocytes), βIII tubulin (neurons) and CNPase (oligodendrocytes) upon different treatments. (C–E) Quantification of the proportion of Hoechst-labeled cells that are positive for (C) GFAP, (D) βIII tubulin, and (E) CNPase. Scale bars represent 50 μm in (A,B). [file Image_1.JPEG]

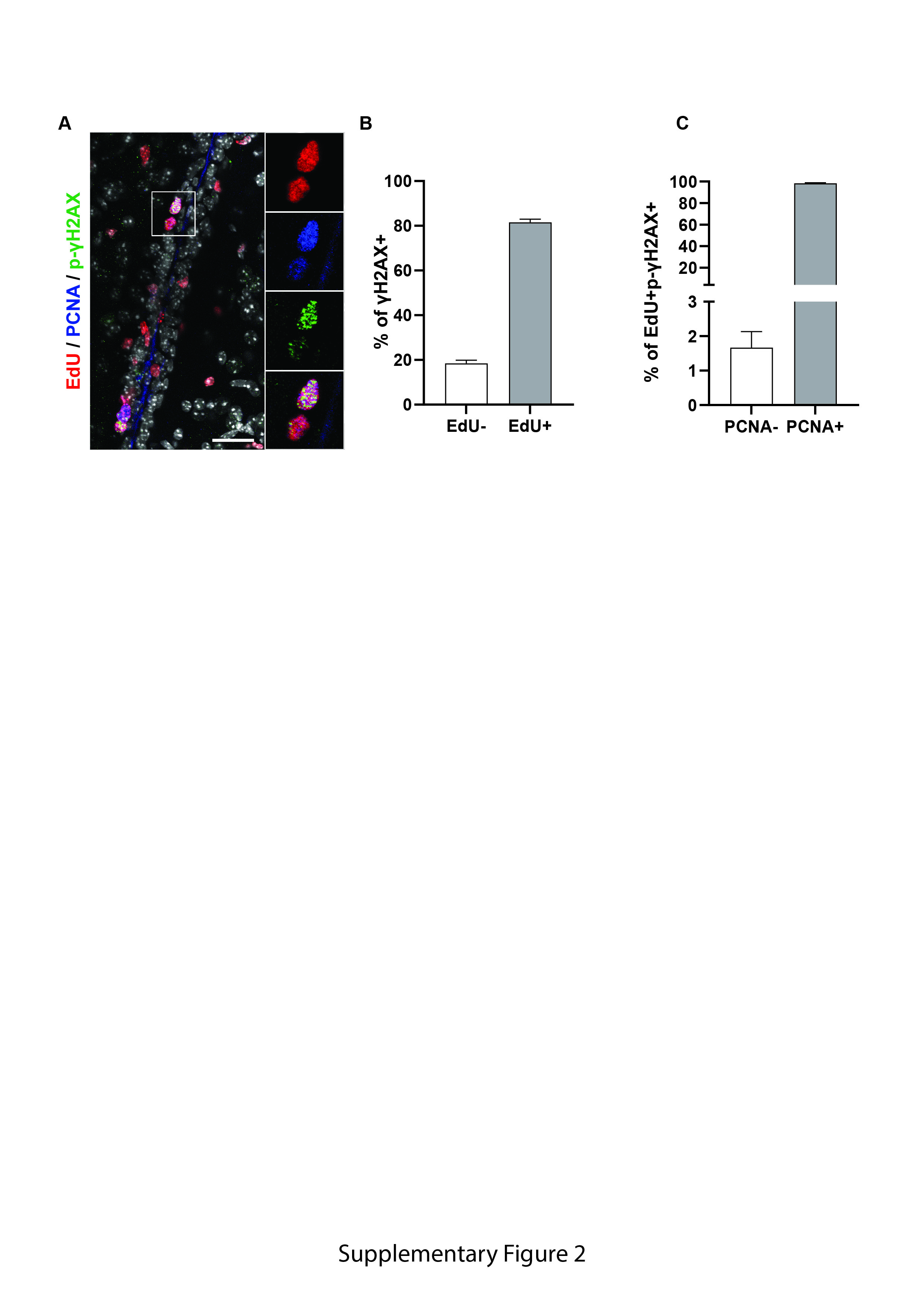

Supplement: Supplementary Figure 2 — (A) Representative micrograph of EdU/p-γH2AX/PCNA staining in the V-SVZ. (B) Quantifications of the p-γH2AX cells labelled with EdU. (C) Quantification of the p-γH2AX + EdU + cells that express PCNA + (actively dividing). Note that virtually all EdU + p-γH2AX + cells are PCNA+. Scale bar represents 50 μm in (A). [file Image_2.JPEG]

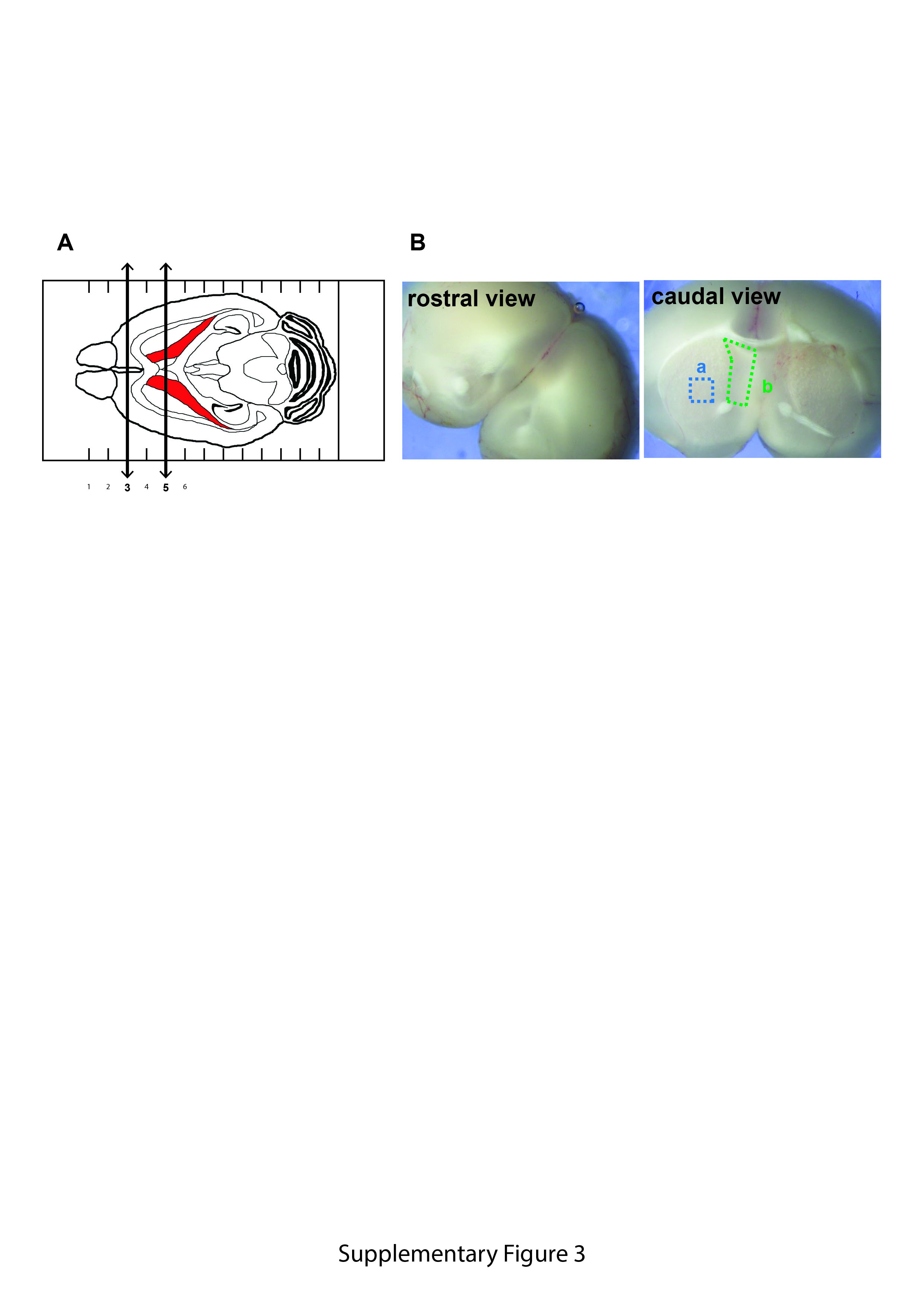

Supplement: Supplementary Figure 3 — (A) Schematic view of the brain slice used for SVZ and striatum microdissections. The outer rectangle represents the brain mold and the lines with arrows represent the site of sectioning with razor blades. (B) Representative micrographs of the slice obtained as seen from the rostral side (left micrograph) and caudal side (right micrograph). The blue dotted square (a) represents region collected for the striatum and the green dotted shape (b) represents the region collected for the V-SVZ. [file Image_3.JPEG]
