# Supplementary material for: Manipulation of EGFR-Induced Signaling for the Recruitment of Quiescent Neural Stem Cells in the Adult Mouse Forebrain
Source: Front Neurosci. 2021 Mar 26;15:621076. doi: 10.3389/fnins.2021.621076 (PMC8032885; doi:10.3389/fnins.2021.621076)
Supplement: Supplementary Table 1 — Plasmid list. [file Table_1.docx]

**Supplementary Table 1**

| Name | Regulatory elements | Gene | Company | Gift of: |
| --- | --- | --- | --- | --- |
| EGFR-CA | LTR | EGFR L858R | Addgene (# 11012) | Matthew Meyerson |
| hGFAP-cre | hGFAP | Cre | Addgene (# 40591) | Albee Messing |
| hGFAPmyrGFP | hGFAP | myrGFP | Addgene (# 22672) | Robert Benezra |

Supplementary Table 1. Plasmid list

**Supplementary Table 2**

| Name | Specie | Company | Dilution |
| --- | --- | --- | --- |
| βIII Tubulin | Mouse | Covance | 1:200 |
| 4EBP1 | Rabbit | Cell Signaling | 1:1000 |
| AKT | Rabbit | Cell Signaling | 1:1000 |
| CNPase | Rabbit | Cell Signaling | 1:200 |
| DCX | Guinea Pig | Chemicon | 1:3000 |
| DCX | Goat | Santa Cruz biotech | 1:250 |
| EGFR | Rabbit | Abcam | 1:100 |
| ERK1/2 | Rabbit | Cell Signaling | 1:3000 |
| GFP | Chicken | Aves Lab | 1:1000 |
| GFAP | Rabbit | Dako Diagnostic | 1:500 |
| GFAP | Mouse | Chemicon | 1:1000 |
| Ki67 | Mouse | BD Biosciences | 1:100 |
| Mash1 | Mouse | BD Biosciences | 1:50 |
| mTOR | Rabbit | Cell Signaling | 1:5000 |
| Olig2 | Rabbit | Chemicon | 1: 250 |
| Sox2 | Rabbit | Chemicon | 1:1000 |
| p4EBP1 | Rabbit | Cell Signaling | 1:500 |
| PCNA | Mouse | BD Bioscience | 1:750 |
| p-AKT | Rabbit | Cell Signaling | 1:2500 |
| p-ERK1/2 | Rabbit | Cell Signaling | 1:250 |
| p-mTOR | Rabbit | Cell Signaling | 1:1000 |
| p-S6 | Rabbit | Cell Signaling | 1:300 |
| S6 | Mouse | Cell Signaling | 1:1000 |
| Secondary Ab (HRP) | Goat, Donkey | Jackson Immuno Research Bio Rad | 1:5000 |
| Secondary Ab (Alexa) | Goat, Donkey | Invitrogen | 1:1000 |

Supplementary Table 2. Antibody list

***Supplementary Figure 1***

**(A)** Representative micrograph of a triple-labelled NSPC culture co-stained for GFAP (first micrograph), βIII tubulin (second micrograph), CNPase+ (third micrograph) and merged (fourth micrograph)

**(B)** Representative micrographs of NSPC cultures stained with GFAP (astrocytes), βIII tubulin (neurons) and CNPase (oligodendrocytes) upon different treatments.

**(C-E)** Quantification of the proportion of Hoechst-labelled cells that are positive for **(C)** GFAP, **(D)** βIII tubulin and **(E)** CNPase.

Scale bars represent 50 µm in **(A)** and **(B)**

***Supplementary Figure 2***

**(A)** Representative micrograph of EdU/p-γH2AX/PCNA staining in the V-SVZ.

**(B)** Quantifications of the p-γH2AX cells labelled with EdU. **(C)** Quantification of the p-γH2AX+EdU+ cells that express PCNA+ (actively dividing).

Note that virtually all EdU+ p-γH2AX+ cells are PCNA+.

Scale bar represents 50 µm in **(A)**

***Supplementary Figure 3***

**(A)** Schematic view of the brain slice used for SVZ and striatum microdissections. The outer rectangle represents the brain mold and the lines with arrows represent the site of sectioning with razor blades.

**(B)** Representative micrographs of the slice obtained as seen from the rostral side (left micrograph) and caudal side (right micrograph). The blue dotted square (a) represents region collected for the striatum and the green dotted shape (b) represents the region collected for the V-SVZ.
